# Development and validation of a two-dimensional pseudorandom balance perturbation test

**DOI:** 10.3389/fnhum.2024.1471132

**Published:** 2024-12-06

**Authors:** Andrew R. Wagner, Sophia G. Chirumbole, Jaclyn B. Caccese, Ajit M. W. Chaudhari, Daniel M. Merfeld

**Affiliations:** ^1^Department of Physical Therapy, School of Pharmacy and Health Professions, Creighton University, Omaha, NE, United States; ^2^Department of Otolaryngology—Head and Neck Surgery, Ohio State University Wexner Medical Center, Columbus, OH, United States; ^3^Mechanical and Aerospace Engineering, Ohio State University, Columbus, OH, United States; ^4^School of Health and Rehabilitation Sciences, Ohio State University, Columbus, OH, United States; ^5^Department of Biomedical Engineering, Ohio State University, Columbus, OH, United States; ^6^Department of Speech and Hearing Science, Ohio State University, Columbus, OH, United States

**Keywords:** vestibular, balance, postural control, multidimensional, perturbation

## Abstract

**Introduction:**

Pseudorandom balance perturbations use unpredictable disturbances of the support surface to quantify reactive postural control. The ability to quantify postural responses to a continuous multidirectional perturbation in two orthogonal dimensions of sway (e.g., AP and ML) has yet to be investigated.

**Methods:**

We developed a balance perturbation paradigm that used two spectrally independent sum of sinusoids signals (SoS_1_, SoS_2_), one for each orthogonal dimension of tilt (roll and pitch), to deliver a two-dimensional (2D) balance perturbation. In a group of 10 healthy adults we measured postural sway during 2D perturbations, as well as for each of the two individual 1D perturbation components.

**Results:**

We found that during 2D perturbations, spectral peaks in the sway response were larger at the perturbed frequencies when compared to (1) the adjacent non-perturbed frequencies and (2) the frequencies contained within the orthogonal, spectrally independent perturbation signal. We also found that for each of the two spectra (SoS_1_, SoS_2_), the magnitude and timing of the sway response relative to the platform disturbance was similar when measured during 1D and 2D conditions.

**Discussion:**

These data support that our novel 2D SoS perturbation test was able to evoke ML and AP postural responses that were (1) specific to the roll and pitch perturbations, respectively, and (2) similar to the responses provoked by individual 1D perturbations.

## Introduction

The vestibular system senses 9 dimensions of information describing head motion and head orientation (3 dimensions each for gravity, linear acceleration, and rotation). This information plays a critical role in the maintenance of postural control, and as a result, balance assessments are commonly employed as a screening tool for vestibular dysfunction (Cohen et al., 2019). Tests of standing balance, i.e., with a fixed-base of support, rely predominantly upon the measurement of spontaneous body sway under the manipulation of sensory feedback [e.g., standing on foam, with eyes closed (Agrawal et al., 2011)]. Among the most common of these tests are (a) a standard 4-condition quiet-stance balance test that is sometimes called the Modified Romberg Test of Standing Balance (Agrawal et al., 2011; CDC, 2021) and (b) the Sensory Organization Test (Nashner and Peters, 1990), which includes anterior-posterior sway-referencing of the motion platform. However, since quiet stance balance tests manipulate balance primarily by degrading sensory feedback, we have no direct knowledge of the exact input stimuli leading to the observed postural sway. As a result, it is challenging to determine if changes in postural sway result from the precision of afferent sensory cues (including vestibular afference) or from the adoption of other exploratory or compensatory postural control strategies (Carpenter et al., 2010). This limitation has motivated the use of perturbed stance balance assessments that measure postural sway in response to a passive unpredictable motion stimulus. Unlike quiet stance, passive pseudorandom balance perturbations allow for the output of the postural control system (i.e., sway) to be described relative to a known, and consistent (across participants), input stimulus (Cenciarini and Peterka, 2006; van der Kooij and Peterka, 2011; Joseph Jilk et al., 2014; Hwang et al., 2016).

Pseudorandom support surface perturbations disturb balance using either discrete or continuous motions that are both unpredictable and independent of postural sway. Discrete perturbations are used to describe the transient postural response to an individual motion stimulus with a set frequency and amplitude (Nashner et al., 1982; Horak et al., 2016), whereas continuous perturbations are used to quantify the steady state response over a range of stimulus frequencies (Peterka, 2002). Continuous pseudorandom perturbations are often created by combining multiple sinusoids [sum-of-sinusoids, SoS (Maki et al., 1994; McAndrew et al., 2010; Sinitksi et al., 2012; Franz et al., 2015; Franz et al., 2017; Kazanski et al., 2020)] or by randomly assigning perturbation velocities to a sequence of numbers [pseudorandom ternary sequence of numbers, PRTS (Cenciarini and Peterka, 2006; Joseph Jilk et al., 2014; Pasma et al., 2015; Wiesmeier et al., 2015; van Kordelaar et al., 2018)]; each method produces an unpredictable motion trajectory secondary to the broad range of spectral components. Although a sinusoidal signal is defined by a formula, and thus, is not truly random, in the early 1960’s Stark and colleagues showed that combining as few as three thoughtfully-selected, non-harmonically related sinusoids yielded a signal that could not be predicted by either naive or experienced human participants (Stark et al., 1961). This has since been confirmed in studies of human postural control (Maki, 1987). Thus, continuous perturbation signals (SoS or PRTS) provide a robust paradigm for studying sensory contributions to balance, due to both (a) the unpredictability of the stimulus, which mitigates feedforward control mechanisms (Peterka and Loughlin, 2004), and (b) the ability to characterize postural control across a wide range of perturbation frequencies (Peterka, 2002; McAndrew et al., 2010).

Studies using 1D PRTS motion stimuli have identified specific patterns of balance dysfunction in patients with well-compensated, chronic vestibular lesions (Peterka, 2002; Peterka et al., 2011; van Kordelaar et al., 2018). As a result of these data, PRTS perturbation paradigms have been increasingly used to study vestibular, as well as other sensory, contributions to balance within a variety of different patient populations (e.g., Pasma et al., 2015; Wiesmeier et al., 2015; Campbell et al., 2022). Yet, although a great deal has been learned from the study of 1D balance perturbations, during naturalistic motion, the vestibular system does not function as a one-dimensional sensor (Wolfe et al., 2021). Instead, the collection of vestibular organs (e.g., semicircular canals and otolith organs) simultaneously sense and encode multiple dimensions of head motion stimuli that each inform unique dimensions of postural sway. As such, a multidimensional perturbed stance balance test may provide a superior method for detecting vestibular impairment by quantifying sensorimotor responses in response to rich, naturalistic multidimensional vestibular stimuli across a range of physiologically relevant frequencies. However, unlike postural responses to discrete multidirectional balance perturbations, which have been well characterized (e.g., Carpenter et al., 1999; Carpenter et al., 2001; Bloem et al., 2002; Grüneberg et al., 2005; Torres-Oviedo et al., 2006; Allum et al., 2008; Willaert et al., 2024), little is known about the steady state postural response to continuous multidimensional perturbations.

**FIGURE 1:**
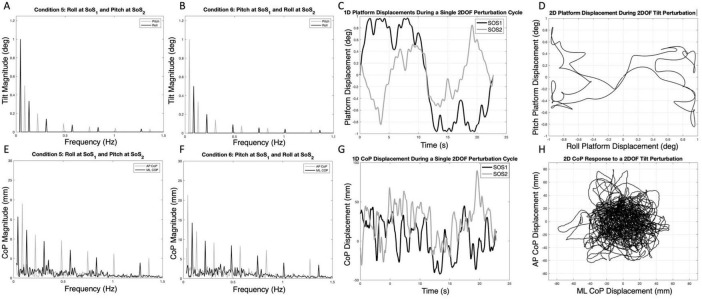
The spectral magnitude of the roll (black) and pitch (gray) perturbation signals are shown for each of the 2D perturbations — Condition 5 **(A)** and Condition 6 **(B)**. The spectral magnitudes of the mediolateral (ML, black) and anteroposterior (AP, gray) center of pressure (CoP) responses are also shown for a single participant in each condition **(E,F)**. The one-dimensional roll and pitch displacement time series of the platform during a two-dimensional perturbation (Condition 5) is shown in **(C)**; here, the SOS_1_ signal (black) is a roll tilt, and the SOS_2_ signal (gray) is a pitch tilt. Exemplar one-dimensional (1D) mediolateral and anteroposterior CoP responses to the SOS_1_ and SOS_2_ perturbations are also shown in **(G)**. The same data from **(C,G)** are shown as two-dimensional plots to demonstrate the two-dimensional travel of the platform **(D)** and the corresponding motion of the CoP for a single cycle of motion **(H)**. Spectral plots [as are shown in **(E,F)**] for each of the 10 participants are provided in Supplementary Figures 2, 3.

Previous groups have studied sagittal plane postural responses to combinations of pitch tilt and fore-aft translation perturbations created using either spectrally independent PRTS signals (with unique fundamental frequencies) (Grüneberg et al., 2005), spectrally independent SoS (Jeka et al., 2004), or using a combination of PRTS and SoS signals (Willaert et al., 2024). Yet, to our knowledge, the ability to quantify postural responses to a multidirectional perturbation in two orthogonal dimensions of sway (e.g., AP and ML) has yet to be investigated. To address this gap in knowledge, we developed and validated a test that uses two spectrally independent SoS signals, one for each direction of tilt (roll and pitch)—to deliver a two-dimensional (2D) balance perturbation that independently perturbed each of two spatially-orthogonal directions of sway [i.e., mediolateral (ML) and anteroposterior (AP), respectively] at different interleaved frequencies (Figure 1). We chose to utilize SoS signals, as opposed to the aforementioned PRTS signal, due to the lower degree of complexity, improved control over the stimulated frequencies, and intuitive expansion to higher dimension perturbations (e.g., 6D). Our hypotheses were (1) that during 2D perturbations, variations in the ML and AP center of pressure (CoP) would be increased primarily at frequencies coinciding with the roll and pitch platform perturbation frequencies, respectively, and (2) that the postural response during a 1D perturbation trial would be similar to the postural response measured during an identical 1D stimulus when delivered as part of a 2D perturbation.

## Materials and methods

### Generation of the sum of sinusoids (SoS) signals

We developed two distinct SoS balance perturbation trajectories (SoS_1_ and SoS_2_) that were orthogonal to each other in time (Figures 1A–D). This was accomplished by selecting two groups of interleaved prime numbers ({1, 3, 7, 13, 19, 29} and {2, 5, 11, 17, 23, 31}) and multiplying them by the fundamental frequency of 0.044 Hz (more precisely, 0.0439453125 Hz, which is a period of 22.76 s yielded by 2048 points at a sampling rate of 90 Hz). The combination of six sinusoids with independent harmonics, the use of unique phase angles for each component, and the long period of each repeating cycle (> 20 s) each add to the unpredictable nature of the SoS perturbation signal. The 90 Hz sampling rate was dictated by the device (Virtualis MotionVR) used. This yielded frequency components at the following approximate frequencies:


f=S⁢o⁢S⁢1[0.044,0.132,0.308,0.571,0.835,1.274]Hz

f=S⁢o⁢S⁢2[0.088,0.220,0.483,0.747,1.011,1.362]Hz

The SoS_1_ and SoS_2_ displacement trajectories (Figures 1C, D), where the subscript refers to the lowest prime multiplier used, were then created by summing sinusoidal signals using the following equations:


SoS(t)1=∑i=1ni=6A1,isin(2πfSoS1,it+∅1,i).

SoS(t)2=∑j=1nj=6A2,jsin(2πfSoS2,jt+∅2,j).

In these equations, respectively, n_*i*_ and n_*j*_ are the number of sinusoids in SoS_1_ and SoS_2_, A_*i*_ and A_*j*_ are the magnitudes of the i’th and j’th sinusoids in SoS_1_ and SoS_2_, f_*i*_ and f_*j*_ are the frequencies of the i’th and j’th sinusoids in SoS_1_ and SoS_2_, φ_*i*_ and φ_*j*_ are the phases of the i’th and j’th sinusoid in SOS_1_ and SOS_2_, and t represents time. This resulted in two SoS time trajectories (SoS_1_ and SoS_2_) that have interleaved, spectrally separated perturbation frequencies (Figures 1A, B). Since the SoS_1_ and SoS_2_ signals started with the prime numbers 1 and 2, the spectral magnitude of displacement at these lowest frequencies were larger than at the subsequent frequencies. Since the vestibular system responds primarily to velocity cues during balance (Jeka et al., 2004), the magnitude and phase (Table 1) of the individual spectral components were chosen to maintain a constant peak velocity (0.28°/s) at each perturbation frequency; this yielded displacement spectra amplitudes that varied as 1/f_*i*_ across the perturbation frequencies. We varied the phase values to keep the peak-to-peak amplitude of the SoS_1_ and SoS_2_ signals within 12.4% of one another (i.e., peak-to-peak amplitudes of 1.94° for the SoS_1_ signal and 1.70° for the SoS_2_ signal) (Supplementary Figure 1). Each signal was used to perturb balance in the roll (mediolateral) and pitch (anteroposterior) dimensions separately (1D perturbations), as well as in novel 2D conditions with the SoS_1_ and SOS_2_ signals being delivered simultaneously, yielding independent frequency spectra for roll and pitch platform tilts.

**TABLE 1:** The frequency (f), amplitude (A), and phase (∅) used to create each of the sum of sines (SoS) time series are shown.

SoS_1_				SoS_2_			
**f_1_**	**A_1_**	**∅_**O**,**1**_**	**∅_**S**,**1**_**	**f_2_**	**A_2_**	**∅_**O**,**2**_**	**∅_**S**,**2**_**
0.0439	1.0000	0	−85.60	0.0879	0.5000	0	91.74
0.1318	0.3333	170	−86.83	0.2197	0.2000	40	269.37
0.3076	0.1429	130	−109.21	0.4834	0.0909	130	274.61
0.5713	0.0769	100	67.07	0.7471	0.0588	220	279.76
0.8350	0.0526	40	213.41	1.0107	0.0435	300	275.07
1.2744	0.0345	160	197.52	1.3623	0.0323	300	282.55

Subscripts (_1,__2_) indicate the spectra used (f_1_ vs. f_2_). ∅_*O*_ represents the original phase value used to create the sinusoidal signals. ∅_*S*_ represents the shifted phase value of the actual signal (calculated using the MATLAB unwrap function) following a time-shift to start both SoS trajectories at a position zero-crossing.

### Test procedures

Postural sway data were collected in ten healthy participants (37 ± 13 years of age). Each participant denied a history of neurological disorders, as well as a history of vestibular or other sensorimotor impairments. A commercially available balance device capable of tilting the support surface ± 12° in the roll and pitch planes (Virtualis MotionVR, Perault, France) was used to perform the platform tilt perturbations. The SoS time series (described above) were first generated using a custom script written in MATLAB (v2020b, Natick, MA) and the resultant CSV files were imported into the Virtualis MotionVR Research software. Two embedded force plates captured center of pressure data at a sampling rate of 90 Hz (Figures 1E–H), which was also the frequency at which the inputs to the motion platform were provided.

Each participant completed the six conditions described in Table 2. The order of the six conditions was randomized across participants to mitigate any unintended order effects. Each condition included seven cycles of perturbations (22.76 s each) and lasted a total of 159.32 s. A narrow stance was used (1–2 cm between medial border of each foot), and each participant stood in stocking feet with arms folded at the chest. Virtual reality (HTC Vive) goggles were worn throughout; however, for the tests reported herein, the goggles displayed only a black screen to remove any visual cues. Participants were also asked to close their eyes during testing. Bose (Quiet Comfort II) over the ear active noise canceling headphones were worn and an audiobook was played throughout each trial to mask any external sounds and to help avoid boredom and keep the participant alert. The audiobook was not synchronized with the stimuli. Prior to each test condition, participants were instructed to stand “upright and relaxed,” and between each of the conditions each participant rested for at least one minute.

**TABLE 2:** The six test conditions are shown.

	Roll perturbation signal	Pitch perturbation signal
Condition 1	SoS_1_	–
Condition 2	SoS_2_	–
Condition 3	–	SoS_1_
Condition 4	–	SoS_2_
Condition 5	SoS_1_	SoS_2_
Condition 6	SoS_2_	SoS_1_

Conditions 1–4 used one-dimensional perturbations, whereas Conditions 5 and 6 used two-dimensional perturbations. SoS_1_ signals contained power at the f_SoS1_ frequencies and the SoS_2_ signals contained power at the f_SoS2_ frequencies.

### Spectral analysis

CoP data were analyzed offline using a custom script in MATLAB. Consistent with existing methods used to analyze responses to PRTS perturbations, the first cycle of each trial was removed prior to the analysis to eliminate transient response components at the onset of the perturbation. The mean of each of CoP signal was first subtracted off, and then a discrete Fourier transform (MATLAB, *fft.m*) was applied to the remaining six cycles of zero-mean data (12,288 data points or 136.52 s) to determine the one-sided power spectra at the perturbed frequencies (f_SoS1_ and f_SoS2_). For each of the six conditions, a metric representing total ML and AP CoP magnitude was calculated by summing the CoP spectral magnitudes separately at the *f*_SoS1_ and *f*_SoS2_ perturbation frequencies. The spectral magnitude of the CoP was also determined at the non-perturbed frequencies (± 0.073 Hz) adjacent to the f_SoS1_ and f_SoS2_ frequencies. This bandwidth was chosen to provide maximum coverage while avoiding overlap with the adjacent perturbation components.


$$T\,o\,t\,a\,l\,M\,L\,C\,o\,P\,M\,a\,g\,n\,i\,t\,u\,d\,e\,a\,t\,f_{S\,o\,S\,1}=\sum_{i=1}^{n\,i=6}M\,L\,C\,o\,P\,M\,a\,g\,n\,i\,t\,u\,d\,e\,\left(f_{S\,o\,S\,1,i}\right)$$

$$T\,o\,t\,a\,l\,M\,L\,C\,o\,P\,M\,a\,g\,n\,i\,t\,u\,d\,e\,a\,t\,f_{S\,o\,S\,2}=\sum_{j=1}^{n\,j=6}M\,L\,C\,o\,P\,M\,a\,g\,n\,i\,t\,u\,d\,e\,\left(f_{S\,o\,S\,2,j}\right)$$

$$T\,o\,t\,a\,l\,A\,P\,C\,o\,P\,M\,a\,g\,n\,i\,t\,u\,d\,e\,a\,t\,f_{S\,o\,S\,1}=\sum_{i=1}^{n\,i=6}A\,P\,C\,o\,P\,M\,a\,g\,n\,i\,t\,u\,d\,e\,\left(f_{S\,o\,S\,1,i}\right)$$

$$T\,o\,t\,a\,l\,A\,P\,C\,o\,P\,M\,a\,g\,n\,i\,t\,u\,d\,e\,a\,t\,f_{S\,o\,S\,2}=\sum_{j=1}^{n\,j=6}A\,P\,C\,o\,P\,M\,a\,g\,n\,i\,t\,u\,d\,e\,\left(f_{S\,o\,S\,2,j}\right)$$

### Frequency response functions

Frequency response functions were used to describe the magnitude and timing of postural responses relative to the tilt perturbations, as a function of frequency. A previously published method was used to estimate the angular displacement of the center of mass (CoM) (Peterka et al., 2018). The CoP data were first filtered using a digital, zero-phase 4th order Butterworth filter with a cut off of 0.47 Hz (Peterka et al., 2018). Consistent with earlier studies (Peterka et al., 2018; Campbell et al., 2022), the height of the CoM (*CoM*_*h*_) was estimated using standard equations (Winter, 1995) according to empirically measured lengths of the (a) leg (medial malleolus to femoral condyles), (b) thigh (femoral condyles to greater trochanter) and, (c) trunk (greater trochanter to glenohumeral joint) segments for each participant. CoM height and the ML and AP CoM displacement (CoM_*ML*_ and CoM_*AP*_) were then used to calculate the roll and pitch sway angles (CoM_θ ,*Roll*_ and CoM_θ,*Pitch*_), respectively.


$$C\,o\,M_{\theta ,R\,o\,l\,l}={\frac{180}{\pi }}^{*}\,\text{asin}\,\left(\frac{C\,o\,M_{M\,L}}{C\,o\,M_{h}}\right)$$

$$C\,o\,M_{\theta ,P\,i\,t\,c\,h}={\frac{180}{\pi }}^{*}\,\text{asin}\,\left(\frac{C\,o\,M_{A\,P}}{C\,o\,M_{h}}\right)$$

The frequency response functions (FRF_*Roll*_ and FRF_*Pitch*_) represent the ratio between the discrete Fourier transform of the estimated CoM angles (*CoM*_θ ,*Roll*_ *or* *CoM*_θ,*Pitch*_) and the platform tilt angles (SoS*_*Roll*_* or SoS_*Pitch*_) at the *f*_SoS1_ and *f*_SoS2_ perturbation frequencies. From the FRFs, we calculated two metrics—the normalized response magnitude (R_Norm_) and phase. R_Norm_ represents the absolute value of the complex numbers from each FRF. When the units of the input and output signals are consistent (degrees), this value is equivalent to the unitless *Gain* parameter (Peterka et al., 2018). Here we refer to this as R_Norm_, rather than *Gain*, to permit a consistent terminology when extending these methods to describe a postural response with units dissimilar to the perturbation stimulus (e.g., degrees of CoM sway per centimeters of platform lateral translation). Phase values, describing the timing of CoM sway relative to the platform perturbation, were calculated using the *unwrap* function in MATLAB which allowed the calculation of phase values beyond ± 180°(Peterka, 2002; Peterka et al., 2018). Similar to the CoP spectral magnitude, we determined R_Norm_ and phase values at individual perturbation frequencies as well as cumulatively across the frequencies included in the SoS_1_ and SoS_2_ perturbation signals.


$$F\,R\,F_{R\,o\,l\,l}\,\left(f_{S\,o\,S\,1}\right)=\frac{D\,F\,T\,\left[C\,o\,M_{R\,o\,l\,l}\,\left(f_{S\,o\,S\,1}\right)\right]}{D\,F\,T\,\left[S\,o\,S_{R\,o\,l\,l}\,\left(f_{S\,o\,S\,1}\right)\right]}$$

$$F\,R\,F_{P\,i\,t\,c\,h}\,\left(f_{S\,o\,S\,1}\right)=\frac{D\,F\,T\,\left[C\,o\,M_{P\,i\,t\,c\,h}\,\left(f_{S\,o\,S\,1}\right)\right]}{D\,F\,T\,\left[S\,o\,S_{P\,i\,t\,c\,h}\,\left(f_{S\,o\,S\,1}\right)\right]}$$

$$F\,R\,F_{R\,o\,l\,l}\,\left(f_{S\,o\,S\,2}\right)=\frac{D\,F\,T\,\left[C\,o\,M_{R\,o\,l\,l}\,\left(f_{S\,o\,S\,2}\right)\right]}{D\,F\,T\,\left[S\,o\,S_{R\,o\,l\,l}\,\left(f_{S\,o\,S\,2}\right)\right]}$$

$$F\,R\,F_{P\,i\,t\,c\,h}\,\left(f_{S\,o\,S\,2}\right)=\frac{D\,F\,T\,\left[C\,o\,M_{P\,i\,t\,c\,h}\,\left(f_{S\,o\,S\,2}\right)\right]}{D\,F\,T\,\left[S\,o\,S_{P\,i\,t\,c\,h}\,\left(f_{S\,o\,S\,2}\right)\right]}$$

### Time domain postural control metrics

In addition to the primary frequency domain analysis, time domain metrics — the root mean square distance (RMSD) and the mean velocity (MVELO) of the CoP — were also calculated separately for AP and ML sway in each of the six test conditions. The RMSD and MVELO metrics provide measures of total CoP sway, which includes sway in response to the perturbation, as well as sway at the non-perturbed frequencies. To calculate the RMSD, the CoP data were first low pass filtered with a 25 Hz cut off using a digital, zero-phase 4th order Butterworth filter (*filtfilt*, MATLAB). The ML and AP RMSD values were calculated by taking the standard deviation of the filtered and zero-mean CoP signals (Karmali et al., 2021). The mean velocity (MVELO) of ML and AP sway was calculated by dividing the CoP path length by the duration of the trial (Prieto et al., 1996).

### Statistical analysis

For the 2D perturbation conditions (Condition 5 and Condition 6), paired *t*-tests (STATA v17, College Station, TX) were used to test for differences in the cumulative ML and AP CoP magnitudes at the SoS_1_ and at the SoS_2_ perturbation frequencies (i.e., f_SoS1_ and f_SoS2_). Prior to performing the tests, the normality of the distributions was assessed using a Shapiro-Wilk test (*p* > 0.05) and by inspecting normal probability plots. In addition to paired *t*-tests, a secondary analysis using linear mixed effect models was run for each condition to determine the difference in AP and ML sway when controlling for the six unique perturbation frequencies. To account for the four comparisons made in each of the above analyses, we report Bonferroni corrected *p*-values (i.e., multiplying each raw *p*-value by 4), with significance set at a corrected *p*-value of *p* < 0.05.

Liner mixed effect models were used to determine the effect of perturbation condition (1D vs. 2D) on the normalized response magnitude (R_Norm_) and phase of the sway response. To account for the repeated measures design, and to adjust for the frequency of the perturbations, each model was first run with perturbation *frequency* and *condition* as fixed effects, and *participant* as a random effect. Each model was then repeated with a *frequency* × *condition* interaction term, and post-hoc F tests of simple effects were run to compare 1D and 2D conditions at each of the individual perturbation frequencies. Acting conservatively, we report uncorrected *p*-values to provide more stringent control over Type II errors that could errantly support our hypothesis of no significant difference between 1D and 2D conditions (i.e., failure to reject the null hypothesis). In a secondary analysis, one-sample *t*-tests were also used to test the null hypothesis that the mean ratio between cumulative R_Norm_ values in the 1D and 2D conditions was equal to 1 (i.e., equivalent sway response). Ratios were constructed for each individual by dividing the cumulative R_Norm_ in the 1D task by the cumulative R_Norm_ in the 2D task for conditions that used identical perturbation stimuli — e.g., the roll response to the SoS_1_ stimulus when delivered during the 1D task (Condition 1) was divided by the roll response to the SoS_1_ stimulus when delivered during the 2D task (Condition 5). Paired *t*-tests were also used to compare the CoP time domain metrics (RMSD and MVELO) between 1D and 2D conditions. As above, we elected not to correct for multiple comparisons to provide a more conservative comparison between conditions.

## Results

### Spectral magnitude of ML vs. AP CoP in the 2D perturbation conditions

**FIGURE 2:**
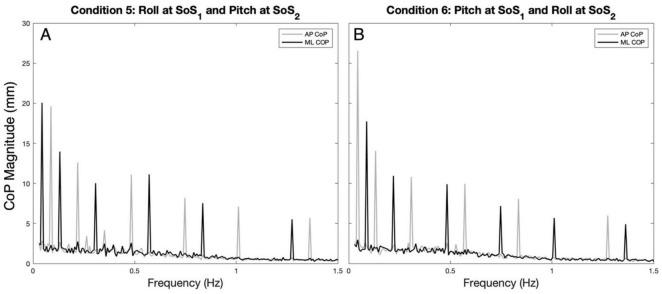
The average (across participants) anteroposterior (AP, gray) and mediolateral (ML, black) CoP spectral magnitudes are shown for the 2D perturbation conditions, Condition 5 **(A)** and Condition 6 **(B)**. The spectral peaks occur at the frequencies (f_SoS1_ and f_SoS2_) of the two unique sum of sinusoids perturbation signals (SoS_1_ and SoS_2_).

Example spectra, time traces and 2D phase plots are shown in Figure 1. The frequency spectra of the platform perturbations are shown in the upper plots (Figures 1A, B) for each of the 2D perturbation conditions (Conditions 5 and 6). Figures 1E, F show example CoP response spectra for a single participant. Figure 2 shows the average ML and AP COP sway spectra across the ten participants for the same 2D perturbation conditions. As can be observed, Figures 1E, F, 2A, B all show the existence of interleaved spectral peaks that coincide with the f_SoS1_ and f_SoS2_ perturbation frequencies.

**FIGURE 3:**
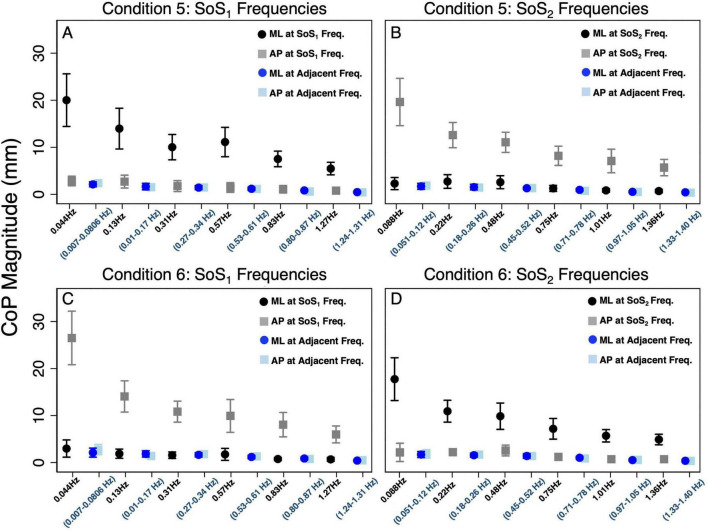
The average (across participants) anteroposterior (AP, gray square) and mediolateral (ML, black circle) CoP spectral magnitudes are shown for each of the stimulated frequencies in the 2D perturbation conditions. **(A,B)** show CoP magnitudes for Condition 5 at the SoS_1_ frequencies and SoS_2_ frequencies, respectively. **(C,D)** show CoP magnitudes for Condition 6 at the SoS_1_ frequencies and SoS_2_ frequencies, respectively. In each plot, the average AP (light blue square) and ML (blue circle) sway at adjacent frequencies is also shown. The adjacent sway response represents the median of the CoP magnitudes surrounding the individual perturbation frequency (± 0.073 Hz). Error bars show +/– 1 SD.

**FIGURE 4:**
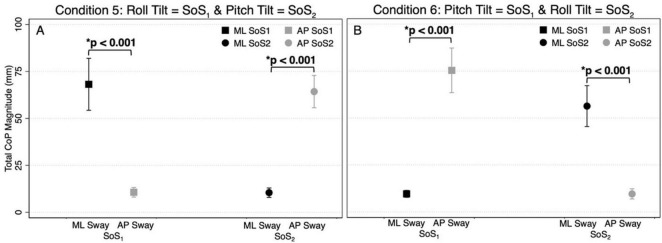
The total CoP magnitudes—summed across frequency — are shown for mediolateral (ML, black) and anteroposterior (AP, gray) postural sway in the 2D perturbation conditions, Condition 5 **(A)** and Condition 6 **(B)**. The left side of each plot shows the mean CoP magnitude (across participants) at the SoS_1_ frequencies (denoted by squares), and the right side of each plot shows the mean CoP magnitude at the SoS_2_ frequencies (denoted by circles). Error bars show ± 1SD. Results of paired *t*-tests comparing the total ML and AP CoP magnitudes are shown.

Figures 3A–D displays the mean and standard deviation (± 1SD) of the spectral magnitude at each of the discrete spectral peaks for the 12 unique perturbation frequencies. These spectral plots show that during 2D perturbations (1) pitch perturbations yielded primarily AP sway at the frequencies of the pitch perturbations with low spectral peaks for ML sway at the same frequencies and (2) roll perturbations yielded primarily ML sway at the frequencies of the roll perturbations with low spectral peaks for AP sway at the same frequencies. To quantitatively describe the CoP response to the specific perturbation spectra (f_SoS1_ and f_SoS2_) in a single metric, we calculated the sum of the CoP magnitude across spectral peaks at the f_SoS1_ frequencies and, separately, at the f_SoS2_ perturbation frequencies (Figure 4). In Condition 5 (where the roll perturbation signal included f_SoS1_ frequencies and the pitch perturbation signal included f_SoS2_ frequencies), as hypothesized, the total ML CoP magnitude was significantly larger than the total AP CoP magnitude [*t*(9) = 14.46, *p* < 0.0001] at the f_SoS1_ frequencies. As hypothesized, the AP CoP magnitude was also significantly larger than the ML CoP magnitude [*t*(9) = 19.79, *p* < 0.0001] at the f_SoS2_ frequencies. In Condition 6 (where the pitch perturbation signal included the f_SoS1_ frequencies and the roll perturbation signal included the f_SoS2_ frequencies), as hypothesized, the ML CoP magnitude was significantly greater than the AP CoP magnitude [*t*(9) = 14.42, *p* < 0.0001] at the f_SoS2_ frequencies. At the f_SoS1_ frequencies, the AP CoP magnitude was significantly greater than the ML CoP magnitude [*t*(9) = 17.11, *p* < 0.0001] as hypothesized (Figure 4).

Figure 3 also shows the differences in CoP magnitude at the perturbed frequencies relative to the CoP sway at the surrounding un-perturbed frequencies (calculated in the same plane of motion by taking the median value of the CoP magnitude at the ten surrounding frequencies). We repeated the above pairwise analyses to also compare the spectral magnitude at the perturbation frequencies to the spectral magnitude at the adjacent non-perturbed frequencies. We found that for each condition, the total spectral magnitude at the perturbed frequencies was significantly greater than the spectral magnitude captured in the same plane at the non-perturbed frequencies (*p* < 0.0001, Figure 3).

Since the difference in total CoP magnitude could be driven by a large difference only at the lowest frequencies (where much of the sway response exists), these comparisons were repeated by running four mixed effect models to determine the differences between the total AP and ML CoP magnitudes, when controlling for the six perturbation frequencies. These models yielded similar findings, showing an increase in the ML sway response at the f_SoS1_ frequencies in Condition 5 (β = 9.58, *p* < 0.001) and the f_SoS2_ frequencies in Condition 6 (β = 7.79, *p* < 0.001). Similarly, the AP CoP magnitude was larger than the ML CoP magnitude at the f_SoS1_ frequencies in Condition 6 (β = 10.96, *p* < 0.001) and the f_SoS2_ frequencies in Condition 5 (β = 8.97, *p* < 0.001). Collectively, these findings are consistent with the graphical data shown in Figures 1–4, and support that during 2D perturbations, (1) sway responses were primarily in the ML direction at the frequencies of the roll perturbation, with minimal AP frequency components at those same frequencies and (2) sway responses were primarily in the AP direction at the frequencies of the pitch perturbation, with minimal ML frequency components at those same frequencies. Individual participant data are provided in Supplementary Figures 2, 3.

### Normalized response magnitude and phase in the 1D versus 2D perturbation conditions

**FIGURE 5:**
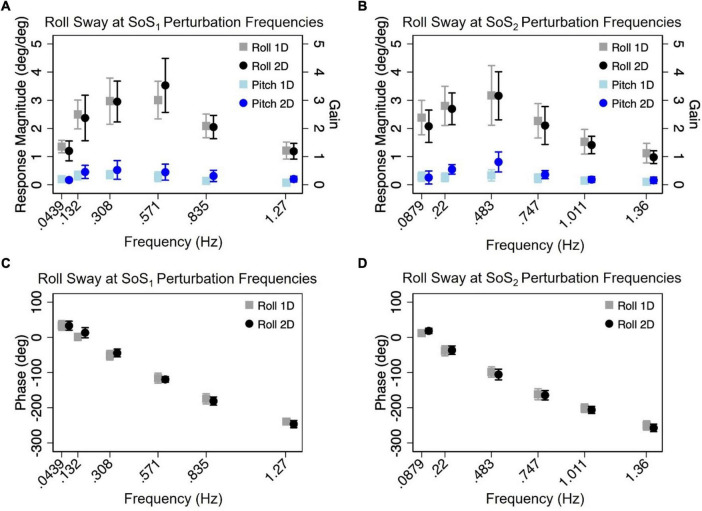
The mean (across participants) normalized response magnitudes **(A,B)** and phases **(C,D)** of the center of mass (CoM) in the roll plane are shown for the 2D (black circle) and 1D (gray square) roll perturbation conditions, at each of the individual f_SoS1_ **(A,C)** and f_SoS2_ **(B,D)** frequencies. The magnitudes of the off-axis pitch plane responses at the roll perturbation frequencies are also shown for 1D (light blue square) and 2D (blue circle) conditions. Error bars show ± 1SD surrounding the mean.

**FIGURE 6:**
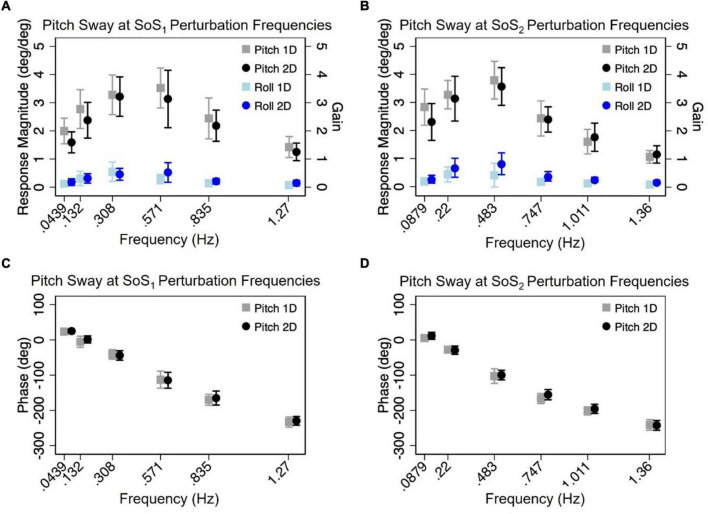
The mean (across participants) normalized response magnitudes **(A,B)** and phases **(C,D)** of the center of mass (CoM) in the pitch plane are shown for the 2D (black circle) and 1D (gray square) pitch perturbation conditions, at each of the individual f_SoS1_ **(A,C)** and f_SoS2_ **(B,D)** frequencies. The magnitudes of the off-axis roll plane responses at the pitch perturbation frequencies are also shown for 1D (light blue square) and 2D (blue circle) conditions. Error bars show ± 1SD surrounding the mean.

Mixed effect models were used to determine if sway responses (R_Norm_ and phase) differed between the 2D and 1D conditions that used identical tilt stimuli with the same frequency spectra (i.e., f_SoS1_ or f_SoS2_) (Figures 5, 6). At the f_SoS1_ frequencies, R_Norm,Roll_ for the 1D condition (Condition 1) was not significantly different from R_Norm,Roll_ in the corresponding 2D condition (Condition 5) (β = 0.26, *p* = 0.762). Similar results were found when comparing R_Norm,Roll_ in Conditions 2 and 6, where the roll tilt signal had power in the f_SoS2_ spectra (β = −0.14, *p* = 0.096). At the f_SoS1_ frequencies, R_Norm,Pitch_ for the 1D condition (Condition 3: 15.43 ± 2.58) was found to be significantly increased relative to the 2D condition (Condition 6: 13.74 ± 2.03) (β = −0.28, *p* = 0.00498). When comparing Condition 4 (1D) and Condition 5 (2D) at the f_SoS2_ frequencies, we did not find a significant difference in R_Norm,Pitch_ values (β = −0.11, *p* = 0.224) (Figures 5, 6). Each of the mixed effect models were then repeated with a *frequency* × *condition* interaction term to test for differences at each of the individual perturbation frequencies (f_SoS1_ or f_SoS2_) (Table 3). Across the 24 tests of simple effects (two conditions × six frequencies × two spectra), we found significant differences between 1D and 2D conditions at only 2 frequencies—at 0.571 Hz for the Roll SoS_1_ stimulus [*F*(1, 99) = 5.96, *p* = 0.0164] and at 0.0879 Hz [*F*(1, 99) = 5.31, *p* = 0.0233] for the Pitch SoS_2_ stimulus (Figures 5, 6). The average differences in R_Norm,Roll_ and R_Norm,Pitch_ between the 1D and 2D conditions across the six frequencies are shown in Table 4. In addition, Figures 5, 6 shows an overlapping of the error bars (± 1SD) for the 1D and 2D conditions at each of the individual perturbation frequencies.

**TABLE 3:** After each mixed effect model, 2D and 1D tasks were compared at each of the individual perturbation frequencies using post-hoc tests of simple effects.

	Roll response		Pitch response	
	**R_Norm_**	**Phase**	**R_Norm_**	**Phase**
**SoS_1_ frequencies**				
0.0439 Hz	0.51, *p* = 0.478	0.02, *p* = 0.879	2.73, *p* = 0.102	0.05, *p* = 0.832
0.132 Hz	0.34, *p* = 0.563	5.62, *p* = 0.0197	2.63, *p* = 0.108	1.19, *p* = 0.278
0.308 Hz	0.01, *p* = 0.935	1.72, *p* = 0.192	0.07, *p* = 0.791	0.22, *p* = 0.638
0.571 Hz	5.96, *p* = 0.0164	0.50, *p* = 0.482	2.54, *p* = 0.114	0.08, *p* = 0.773
0.835 Hz	0.03, *p* = 0.854	1.7, *p* = 0.195	1.15, *p* = 0.287	0.64, *p* = 0.425
1.27 Hz	0.02, *p* = 0.894	2.13, *p* = 0.147	0.51, *p* = 0.479	0.38, *p* = 0.538
**SoS_2_ frequencies**				
0.0879 Hz	2.18, *p* = 0.143	1.31, *p* = 0.255	5.31, *p* = 0.0233	1.85, *p* = 0.177
0.22 Hz	0.25, *p* = 0.621	0.07, *p* = 0.79	0.38, *p* = 0.541	0.14, *p* = 0.711
0.483 Hz	0.01, *p* = 0.949	1.55, *p* = 0.217	0.97, *p* = 0.326	0.4, *p* = 0.526
0.747 Hz	0.61, *p* = 0.439	0.27, *p* = 0.604	0.03, *p* = 0.866	5.68, *p* = 0.019
1.011 Hz	0.29, *p* = 0.588	0.83, *p* = 0.363	0.49, *p* = 0.487	1.56, *p* = 0.214
1.36 Hz	0.46, *p* = 0.497	1.55, *p* = 0.216	0.14, *p* = 0.710	0.06, *p* = 0.812

Each cell shows the F score [*F*(1, 99)] and corresponding *p*-values. Secondary to our *a priori* hypothesis of no significant difference between conditions (i.e., failure to reject the null hypothesis), we report uncorrected *p*-values to provide more stringent control over Type II errors that could errantly support our hypothesis. Shaded cells indicate significance at *p* < 0.05. R_Norm_ = normalized response magnitude.

**TABLE 4:** For each participant, differences in the normalized response magnitude (R_norm_) and CoM phase were calculated between the 2D and 1D conditions at each of the six perturbation frequencies.

	Average R_Norm_ difference (deg/deg)	Average phase difference (deg)
Roll—SoS_1_	0.027 ± 0.16	0.05 ± 2.99
Roll—SoS_2_	−0.14 ± 0.19	−2.30 ± 5.08
Pitch—SoS_1_	−0.28 ± 0.28	1.99 ± 4.00
Pitch—SoS_2_	−0.11 ± 0.28	3.87 ± 7.00

The average difference across frequencies (and standard deviation) are reported for each frequency spectra (SoS_1_ and SoS_2_) and for each dimension of sway (roll and pitch). Negative values indicate greater values for the 2D compared to 1D condition.

**FIGURE 7:**
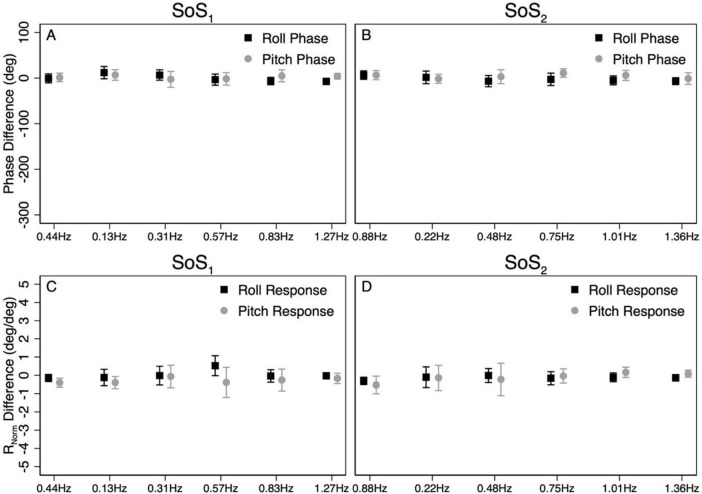
The average difference in CoM phase between 1D and 2D conditions for the SoS_1_ **(A)** and SoS_2_ **(B)** perturbation stimuli are shown. For each participant, the differences in phase between the 2D and 1D conditions were calculated at each of the six perturbation frequencies. The average phase difference was then calculated by taking the average across the six perturbation frequencies. To demonstrate the magnitude of the phase differences relative to the calculated phase values, the scaling of the *y*-axis was matched to Figures 5, 6. Average differences in the normalized response magnitude (R_Norm_) at each frequency were also calculated using identical methods **(C,D)**. Error bars show ± 1SD.

The above analyses were repeated to test for differences in CoM phase angles between each pair of 1D and 2D conditions. Overall, differences in the roll CoM phase angle were minimal for each frequency spectra (f_SoS1_: Mean Difference = 0.05° ± 2.99, f_SoS2_: Mean Difference = −2.30° ± 5.08°) (Figure 7 and Table 4). We did not identify a significant difference in phase between conditions 5 and 1 (β = 0.0495, *p* = 0.981) or between conditions 2 and 6 (β = −2.30, *p* = 0.307), when controlling for perturbation frequency. Similar to the roll plane, differences in the pitch CoM phase angle between the 2D and 1D conditions were also minimal (SoS_1_: Mean Difference = 1.99° ± 4.00, SoS_2_: Mean Difference 3.87° ± 7.00°) (Figure 7 and Table 4). The differences in phase between conditions 3 and 6 (β = 1.99, *p* = 0.418) was not significant, but we did observe a borderline significant difference in phase between conditions 4 and 5 (β = 3.872, *p* = 0.043). When the analyses were repeated with a *frequency* × *condition* interaction term, we identified only 2 of 24 significant differences — at 0.132 Hz for the Roll SoS_1_ stimulus [*F*(1, 99) = 5.62, *p* = 0.0197] and at 0.747 Hz [*F*(1, 99) = 5.68, *p* = 0.019] for the Pitch SoS_2_ stimulus. Individual participant data are provided in Supplementary Figures 4–11.

To further determine the similarities between the response to 1D trials and the response to individual 1D components of 2D trials, we also calculated the ratio between the 1D and 2D cumulative R_Norm_ values for each participant. In three out of the four ratios (Group Mean[95% CI]) we did not have sufficient evidence to reject the null hypothesis that the responses to the 1D and 2D stimuli were not different from one another (i.e., the 95% confidence intervals surrounding the mean included the value 1.00) {Roll SoS_1_ : 0.993 [0.95–1.04], *t*(9) = −0.35, *p* = 0.73, Roll SoS_2_ : 1.07 [0.99, 1.15], *t*(9) = 2.1, *p* = 0.065, Pitch SoS_2_ : 1.06 [0.97–1.15], *t*(9) = 1.42, *p* = 0.19}. However, similar to the results of the mixed effect model, for the SoS_1_ stimulus, the pitch response to the 1D condition (Condition 3) was significantly larger than the response to the 2D condition (Condition 6) {1.13 [1.02, 1.23], *t*(9) = 2.8, *p* = 0.021}.

### Time domain measures in the 1D vs. 2D perturbation conditions

**FIGURE 8:**
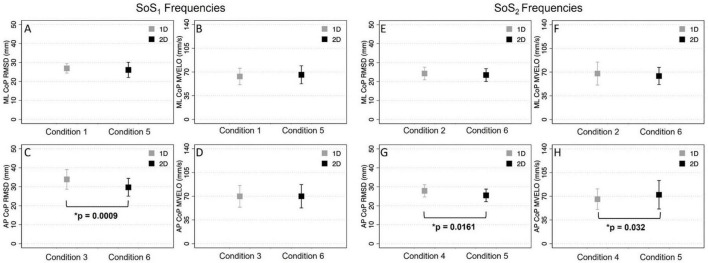
The root mean square distance (RMSD) and mean velocity (MVELO) of the center of pressure (CoP) are shown for each 1D (gray) and 2D (black) condition. Each plot shows the group mean ± 1SD. The two left columns **(A,B,C,D)** show comparisons for conditions that included a perturbation stimulus with power at the SoS_1_ frequencies. The two right columns **(E,F,G,H)** show comparisons for conditions that included a perturbation stimulus with power at the SoS_2_ frequencies. Significant differences (*p* < 0.05) are shown.

The ML RMSD of the CoP [*t*(9) = 1.22, *p* = 0.252] was not significantly different in Condition 1 (1D SoS_1_ roll perturbation) compared to Condition 5 (2D SoS_1_ roll perturbation) (Table 5). Similarly, no difference was seen when comparing the ML RMSD in Condition 2 (1D SoS_2_ roll perturbation) and Condition 6 (2D SoS_2_ roll perturbation) [*t*(9) = 2.00, *p* = 0.076]. We did, however, observe a significant increase in the AP RMSD for the 1D compared to 2D SoS_1_ pitch perturbation conditions [Condition 3 vs. Condition 6: *t*(9) = 4.83, *p* = 0.0009]. A lesser (9.9%), but still significant difference in AP RMSD was also found between the 1D and 2D trials that used SoS_2_ pitch perturbations [Condition 4 vs. Condition 5: *t*(9) = 2.95, *p* = 0.0161] (Figure 8).

**TABLE 5:** The root mean square distance (RMSD) and the mean velocity (MVELO) of the mediolateral (ML) and anteroposterior (AP) center of pressure (CoP) are shown for each of the six perturbation conditions.

	Root mean square distance (mm)		Mean velocity (mm/s)	
	**ML**	**AP**	**ML**	**AP**
Condition 1: Roll SoS_1_	26.94 ± 2.52	11.61 ± 1.90	63.40 ± 12.14	33.89 ± 9.01
Condition 2: Roll SoS_2_	24.26 ± 3.33	10.81 ± 2.27	67.85 ± 16.93	34.64 ± 10.64
Condition 3: Pitch SoS_1_	9.57 ± 2.88	33.87 ± 5.19	26.68 ± 6.70	69.82 ± 16.00
Condition 4: Pitch SoS_2_	10.28 ± 3.98	27.87 ± 3.21	27.16 ± 8.27	65.74 ± 15.20
Condition 5: Roll SoS_1_ + Pitch SoS_2_	26.08 ± 3.99	25.49 ± 3.30	65.84 ± 13.29	72.23 ± 21.03
Condition 6: Roll SoS_2_ + Pitch SoS_1_	23.45 ± 3.36	29.71 ± 4.69	64.26 ± 12.67	69.97 ± 17.26

SoS_1_, sum of sinusoids time series with power at f_SoS1_ frequencies; SoS_2_, sum of sinusoids time series with power at the f_SoS2_ frequencies.

When comparing the mean CoP velocity (MVELO), we found that the ML MVELO was similar in the 1D and 2D conditions for both the SoS_1_ [*t*(9) = −1.6, *p* > 0.145] and SoS_2_ [*t*(9) = 1.41, *p* > 0.193] roll perturbation conditions (Table 5 and Figure 8). When comparing the AP MVELO, a small (7.75%) but significant increase in sway velocity was found for 2D relative 1D trials that used SoS_2_ [*t*(9) = −2.53, *p* = 0.032], but not SoS_1_ [*t*(9) = −0.061, *p* > 0.953] pitch perturbations.

## Discussion

In support of our primary hypotheses, we report two primary findings: (1) During continuous 2D perturbations, changes in the ML and AP center of pressure (CoP) were increased primarily at the frequencies coinciding with the roll and pitch platform perturbation frequencies, respectively. (2) During 2D perturbations, the postural responses to each 1D component were similar to the responses measured during individual 1D tasks (i.e., roll or pitch tilts in isolation). These findings support that the human postural system can independently respond in two orthogonal sway dimensions when two spectrally independent SoS perturbations are provided in the roll and pitch dimensions.

Our findings are consistent with earlier studies of quiet stance balance, as well as discrete multidirectional balance perturbations, which identified independent control strategies for roll and pitch balance responses (Winter et al., 1996; Carpenter et al., 1999). Winter et al. (1996) showed that during quiet stance, AP sway occurred primarily through dorsiflexion/plantarflexion at the ankle, whereas ML sway occurred as a result of proximal motion at the hip (Winter et al., 1996). Although the present study is the first to combine *continuous* roll and pitch platform rotations, previous studies have similarly identified independent organization of roll and pitch balance responses during *discrete* multidirectional platform perturbations (Carpenter et al., 2001; Carpenter et al., 2001; Grüneberg et al., 2005; Allum et al., 2008). Specifically, Grüneberg et al. (2005) showed that similar to quiet stance, roll plane responses to discrete multidirectional platform tilts were controlled primarily at the trunk (i.e., hip strategy), whereas pitch plane responses were controlled primarily at the ankle (i.e., ankle strategy) (Grüneberg et al., 2005). By using a continuous pseudorandom motion stimulus, our data adds to this body of literature by showing that roll and pitch balance responses are separable during a continuous, steady state response, and that the sway response is specific to the spectral components that constituted the roll and pitch balance perturbations. Based on these data, we posit that during multidimensional motion, individuals having an intact postural control system are capable of generating separable, largely independent, responses to at least two dimensions of balance disturbance (i.e., roll and pitch). We also found that the responses generated during 2D conditions were similar to those generated during separate administration of the individual 1D components. Specifically, the CoM response (R_Norm_ and phase), as well as the time domain CoP metrics (RMSD and MVELO), were similar when measured during 1D (i.e., roll or pitch tilt delivered in isolation) and 2D (i.e., roll and pitch tilts delivered simultaneously) perturbations. The shapes of the frequency response functions for the 2D perturbation conditions were also qualitatively similar to past 1D PRTS and 1D SoS studies (Peterka, 2002; Peterka et al., 2011; Joseph Jilk et al., 2014; Wiesmeier et al., 2015), as well as being quantitatively similar to our own 1D trials (Figures 5, 6). Thus, the two-dimensional data gathered from the 2D trial appears to be largely equivalent to the one-dimensional data gathered from two separate 1D trials.

Our findings are consistent with a recent study of two-dimensional perturbations delivered in the sagittal plane (Lippi et al., 2023). Lippi et al. (2023) showed that in the majority of comparisons (∼80%), the frequency response functions describing the response to a primary perturbation (e.g., pitch platform tilt) were unchanged when a concurrent, yet spectrally independent, fore-aft translation stimulus was added; the same was true in regard to the postural response to fore-aft translations during concurrent pitch tilts of the platform. Our data, however, suggest that in addition to showing that postural responses are similar during 2D (roll + pitch tilts) and 1D (roll or pitch tilt only) perturbations, the response to the second stimulus also evoked a specific postural response, as indicated by separable ML and AP CoP responses in the 2D perturbation conditions.

However, similar to Lippi et al. (2023) we did identify several exceptions to this finding (4 out of 48 pairwise comparisons or 8.3%, Table 3). The AP sway response during 1D trials (i.e., Conditions 3 and 4) was often greater than the AP sway response to identical pitch stimuli when delivered during 2D trials (i.e., Conditions 5 and 6). Due to our use of a narrow base of support — which creates a preferential challenge for ML as compared to AP postural control — the pitch response may have been reduced during 2D trials due to a greater need to compensate for the orthogonal roll stimulus. Future studies should consider testing of a widened base of support or scaling the amplitude of the roll and pitch tilt stimuli to accommodate for differences in ML and AP stability in narrow stance. However, while the above differences were significant (*p* < 0.05), we highlight that: (1) we opted not to correct for multiple comparisons to avoid obfuscating small differences between 1D and 2D tasks, (2) the magnitude of the differences between 1D and 2D trials (< 15%) was not substantial relative to past studies of inter-trial variability during quiet stance (Worthen-Chaudhari et al., 2018), and (3) this difference was influenced by a participant who demonstrated a cumulative response during the 1D SoS_1_ pitch trial that was nearly two standard deviations from the sample mean. Furthermore, using a binomial exact calculation, the 95% confidence interval is 2.3 to 20.0%, which includes 5% as would be expected in the absence of a statistical effect given that we used the standard statistical criterion of *p* < 0.05. Thus, despite small differences in the pitch response, we posit that 2D SoS balance perturbations represent a promising strategy for characterizing individual dimensions of balance dysfunction (roll vs. pitch stability).

Secondary to the multimodal nature of the vestibular system, multidimensional perturbations may also provide a novel methodology for probing specific contributions of the vestibular system to postural control. The postural response to a 1D PRTS perturbation has previously been predicted by a simulation model driven primarily by afferent sensory feedback (van der Kooij and Peterka, 2011). Empirical data also support that 1D PRTS perturbations elicit specific patterns of balance dysfunction in individuals with chronic, well compensated vestibular pathologies (Peterka, 2002; van der Kooij and Peterka, 2011; van Kordelaar et al., 2018). However, a single 1D test is inherently constrained in that it cannot characterize the influence of vestibular pathology on postural control in the remaining unperturbed dimensions of head motion. However, to our knowledge, existing PRTS, as well as SoS, balance perturbations have thus far only included perturbations delivered along a single dimension of motion. Thus, whereas existing state-of-the-art 1D assessments can broadly identify vestibular impairment, they cannot separate and/or quantify the influence of impairments in different vestibular modalities (e.g., roll vs. pitch motion) on postural control during a multidimensional balance perturbation. In the present study, we intentionally selected test parameters that we expected, based upon these earlier 1D PRTS studies, would prioritize the use of vestibular feedback for postural control (e.g., pseudorandom stimulus, eyes closed, ∼2° peak-to-peak tilt amplitude). Moreover, the multidimensional nature of the 2D perturbation, being unpredictable in time, direction, and dimension is likely to have also prioritized the use of vestibular feedback. Thus, while acknowledging the multisensory contributions to balance responses, we suggest that the observed sway response was likely driven in large part by vestibular feedback due to the nature of the test conditions.

Additional evidence to support the relevance of vestibular feedback during 2D perturbations comes from a recently published study that compared tests of vestibular precision with postural sway provoked by a two-dimensional endogenous platform perturbation; unlike the pseudorandom SoS stimulus used here, this 2D perturbation was achieved by sway referencing the support surface in both the roll and pitch dimensions (Wagner and Merfeld, 2023). In this earlier study, roll-tilt vestibular perceptual thresholds showed a stronger correlation with the RMSD of the CoP in the 2D sway referenced condition when compared to a standard “eyes closed, on foam” quiet stance assessment (Wagner et al., 2023). A previous study also showed only weak correlations between vestibular thresholds and a 1D sway referenced pitch perturbation (Karmali et al., 2021).

While these 2D perturbation data are promising, additional studies are needed to test the hypothesized reliance upon vestibular feedback during 2D balance perturbations. Of particular interest will be the determination of whether specific dimensions of balance impairment (e.g., roll instability) are associated with sensory impairment in the same dimension (e.g., impaired perception of roll motion cues). Future studies should also determine if individuals with vestibular dysfunction, in contrast to individuals with an intact postural control system, might display preferential impairments during 2D relative to 1D perturbations.

It is, however, worth noting, that while we presume independent control of pitch and roll postural sway, the specific motor control strategies that led to the observed sway responses cannot be determined from our data without corresponding measures of muscle activation (e.g., electromyography). Both human and animal studies have shown that a variety of sensorimotor behaviors can be accomplished through the recruitment of only a few specific muscle synergies (i.e., goal directed patterns of muscle activity) (Macpherson, 1988; Macpherson, 1994; Torres-Oviedo et al., 2006; Chvatal et al., 2011; Chvatal and Ting, 2013; Koehn et al., 2022). These data lead us to speculate that the independent organization of roll and pitch postural responses that we observed may result from the use of at least two distinct muscle synergies that act individually to control the response to roll and pitch perturbations. Although prior work has suggested a distal to proximal organization of balance response (Nashner et al., 1982), the presence of early stretch responses at the hip during roll perturbations (Carpenter et al., 1999; Bloem et al., 2000; Bloem et al., 2002) instead suggests unique, but complementary roles for distal and proximal motor control strategies. Moreover, Torres-Oliviero et al. (2006) showed that during multidirectional platform translations, proximal muscles responded to ML translations with a latency similar to what was measured at the distal ankle during AP translations (Torres-Oviedo et al., 2006). Thus, during multidimensional perturbations, the independent control of pitch and roll postural responses may be facilitated by the use of different muscle synergies (e.g., hip vs. ankle) that, based upon sensory feedback, served to independently control roll and pitch sway. Similarities in the response to the 1D and 2D conditions could also indicate that the same muscle synergies are used to respond to both 1D and 2D perturbations. Future studies should test this hypothesis by replicating our paradigm while recording lower extremity EMG data.

Although both SoS and PRTS methods generate pseudorandom signals—i.e., signals that appear random to naive individuals (Peterka, 2002) − and have been used to generate multidimensional balance perturbations (Pasma et al., 2012; Engelhart et al., 2016; Lippi et al., 2023), we suggest that SoS stimuli may hold advantages when implementing multidimensional balance perturbations. A fundamental characteristic of a PRTS signal is the inclusion of spectral harmonics at all odd multiples of the fundamental frequency (Peterka, 2002). In contrast, SoS trajectories are generated by selecting a predetermined list of frequencies based upon (a) the fundamental frequency, which is defined by the duration of the motion trajectory and (b) a series of prime numbers; this ensures that the harmonics included in the SoS signal are independent (i.e., not multiples of one another) and that each signal contains full cycles of each component.

We also highlight several additional limitations of our study. The generalizability of our findings is limited due to our choice to include only healthy asymptomatic adults. Our data therefore describe the specific capabilities of an intact postural control system. Additional studies will be needed to determine if individuals with vestibular impairment show similar responses during 2D perturbations. Such studies should also determine if specific patient populations show preferential changes in response to individual dimensions of balance perturbations, as such changes may represent specific impairments of the semicircular canals or otolith organs. Similarly, our conclusion of a vestibular driven response to 2D motion is dependent on inference from past 1D studies, and thus should be tested in individuals with confirmed vestibular impairment. Also, while the postural responses were similar between 1D and 2D conditions, we did find several small differences in the pitch response. Future studies should investigate if the identified differences in performance are real or are the result of variability inherent to our moderate sample size. A final limitation of the study was the description of the postural response from CoP data, rather than from alternative methods such as optical motion capture and/or EMG. To help address this limitation, we verified our results with an independent source of postural sway data captured from the HTC Vive head mounted display (HMD). These data, including the methods used for processing, are provided in Supplementary Figures 12–23. Overall, we found that balance quantified via the HMD were consistent with the CoP data that we focus on herein.

## Conclusion

We found that during continuous 2D SoS perturbations, changes in the ML and AP center of pressure (CoP) were increased primarily at the frequencies coinciding with the roll and pitch platform perturbation frequencies, respectively. In addition, we found that during 2D perturbations, the postural responses to each 1D component were similar to the responses measured during individual 1D tasks (i.e., roll or pitch tilts in isolation). These findings support that healthy adults can independently respond in two orthogonal sway dimensions when two spectrally independent SoS perturbations are provided in the roll and pitch dimensions.

## Data Availability

The raw data supporting the conclusions of this article will be made available by the authors, without undue reservation.
