# Supplementary material for: Development and validation of a two-dimensional pseudorandom balance perturbation test
Source: Front Hum Neurosci. 2024 Dec 6;18:1471132. doi: 10.3389/fnhum.2024.1471132 (PMC11659295; doi:10.3389/fnhum.2024.1471132)
Supplement: Supplementary file 1 [file Data_Sheet_1.docx]

**Supplementary Figure 1**

**
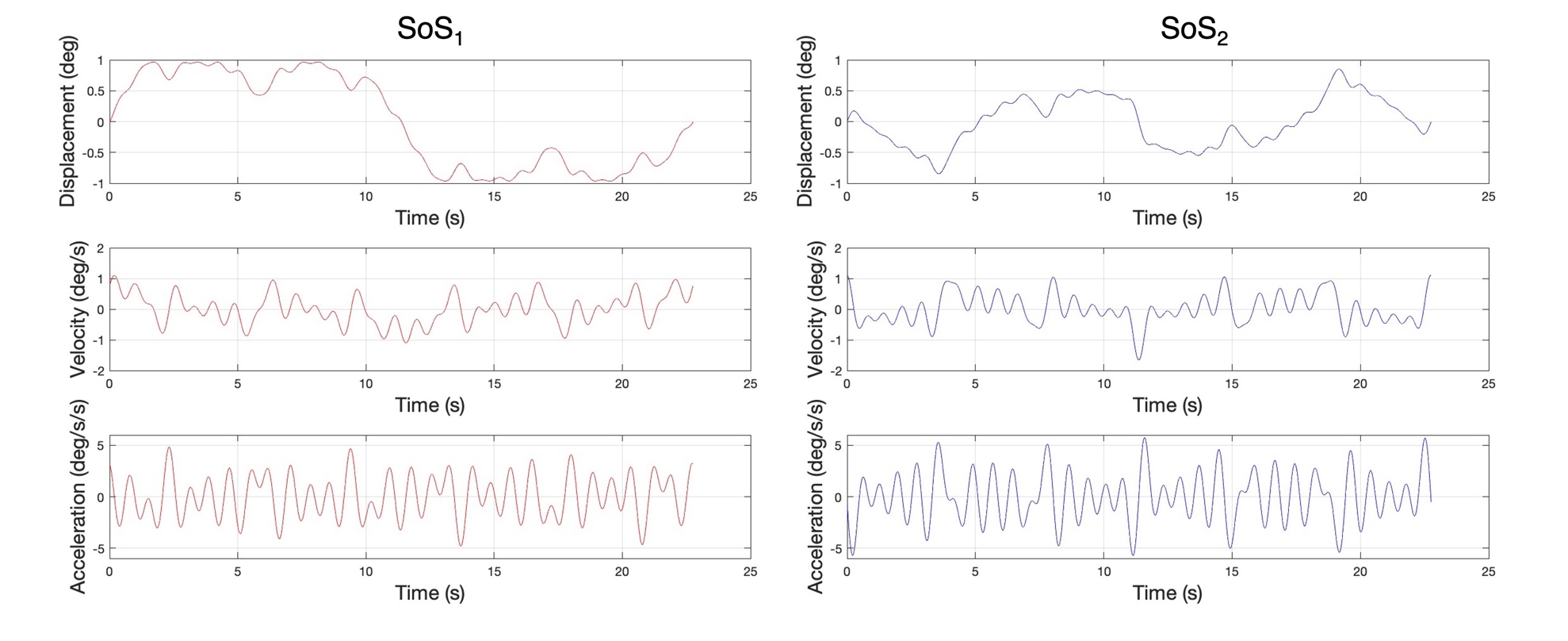
**

**Supplementary Figure 2**

**
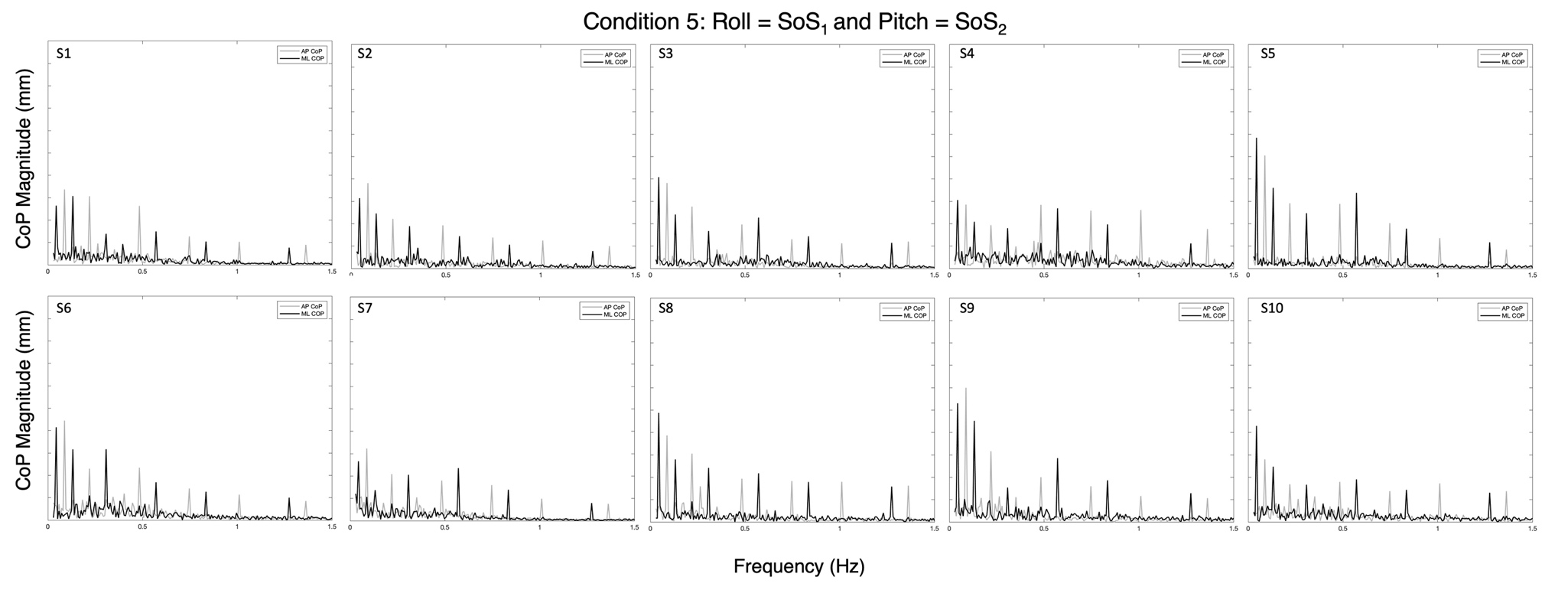
**

**Supplementary Figure 3**

**
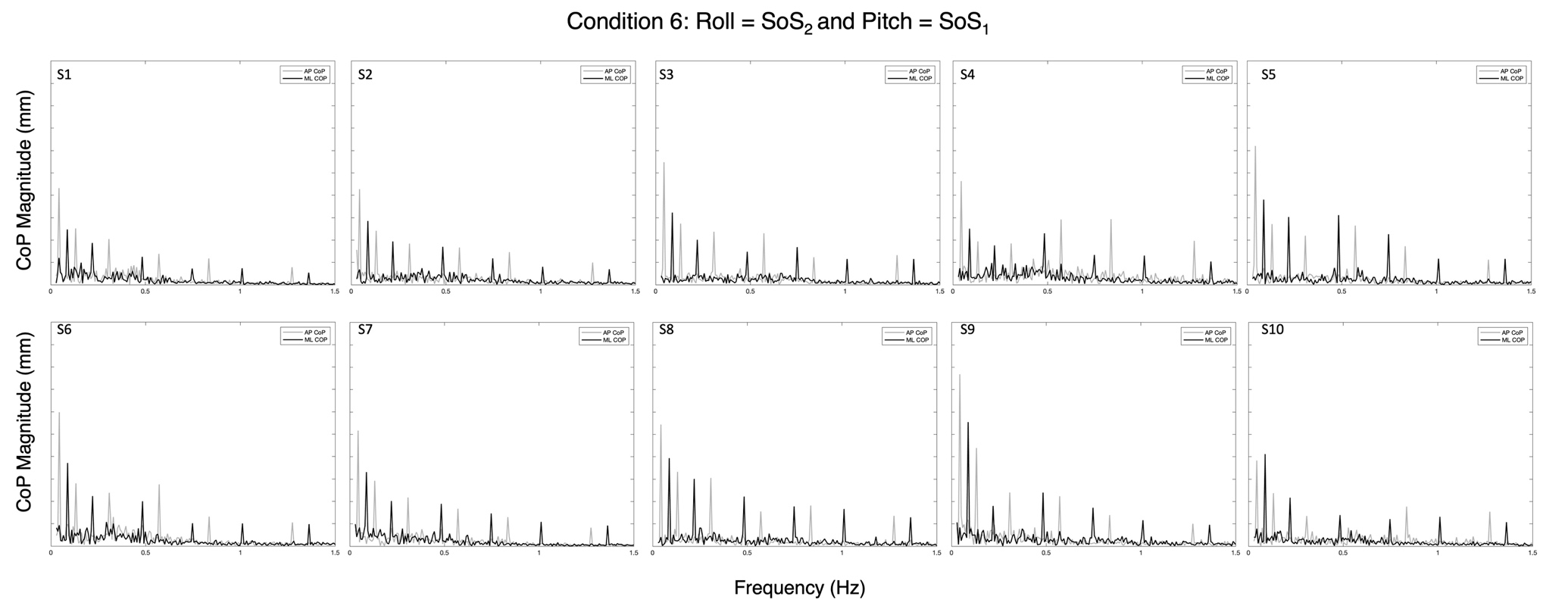
**

**Supplementary Figure 4**

**
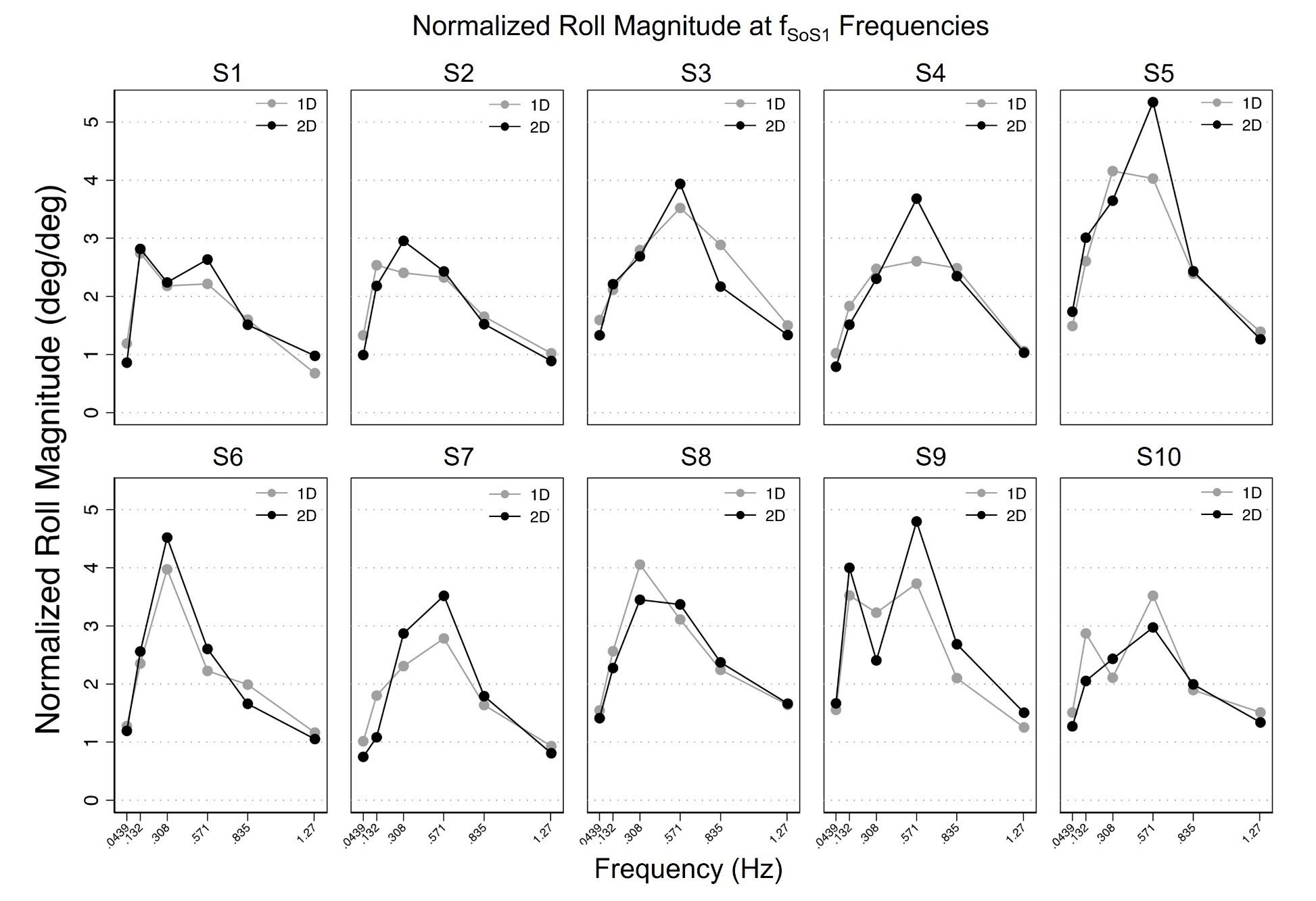
**

**Supplementary Figure 5**

**
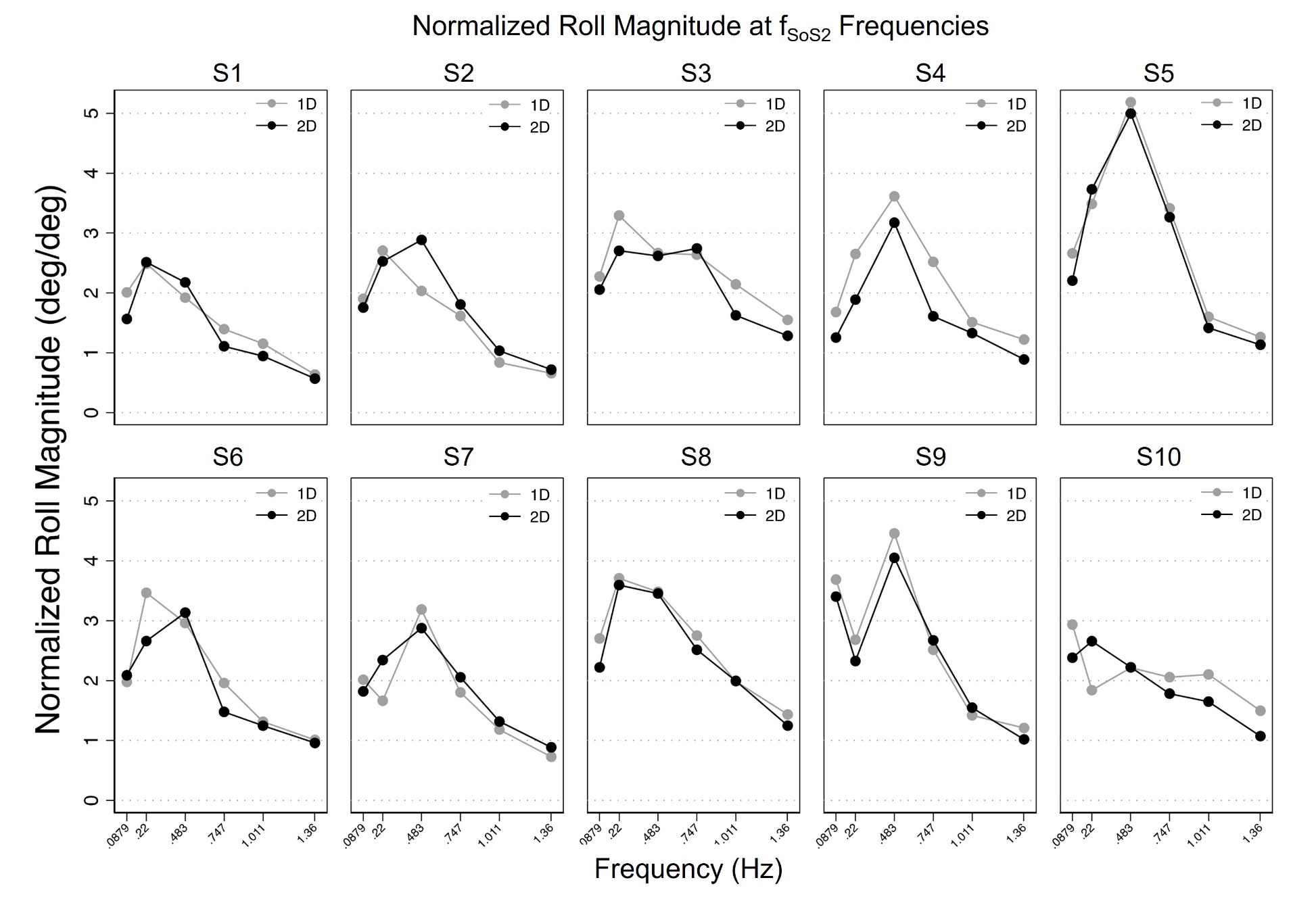
**

**Supplementary Figure 6**

**
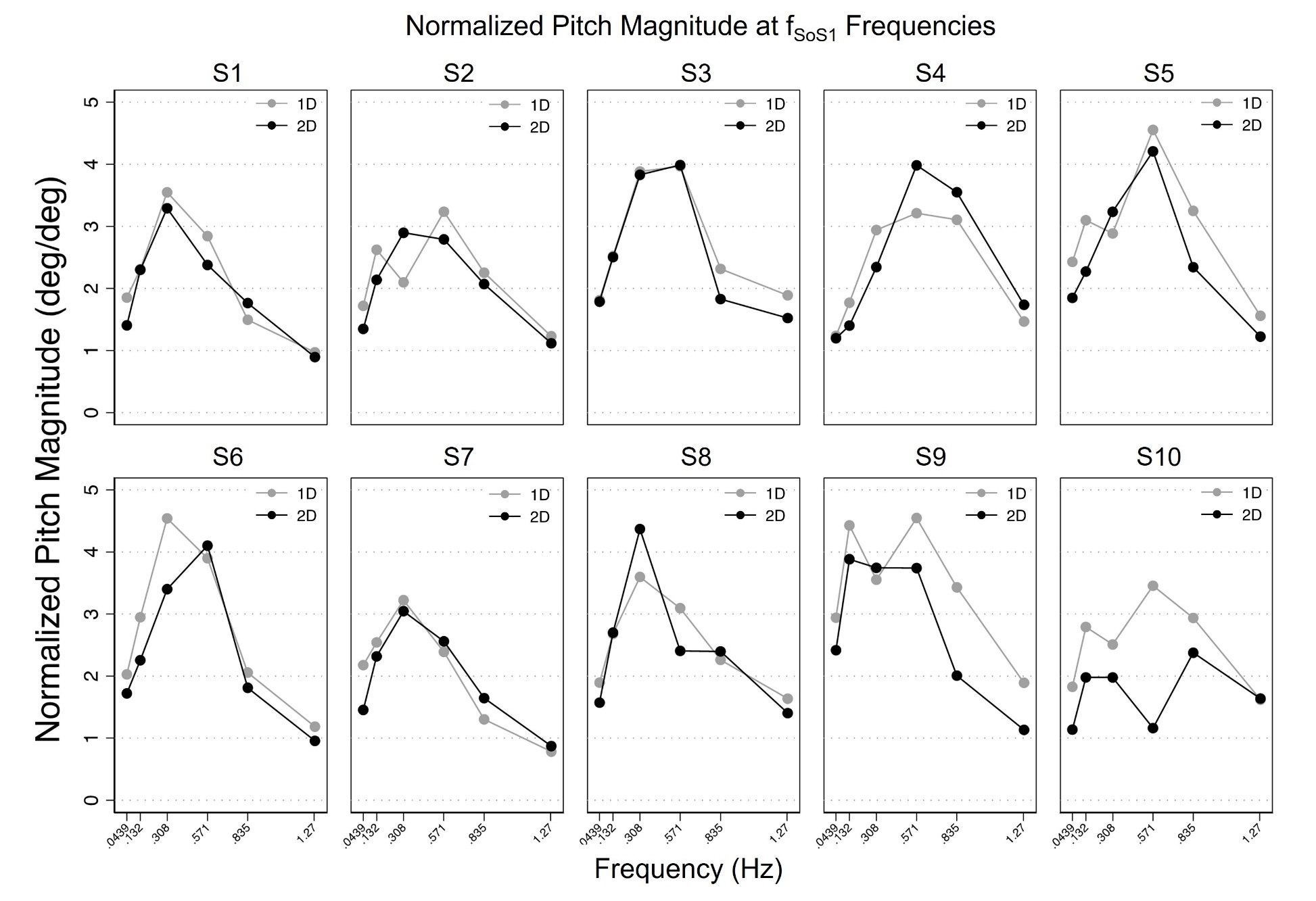
**

**Supplementary Figure 7**

**
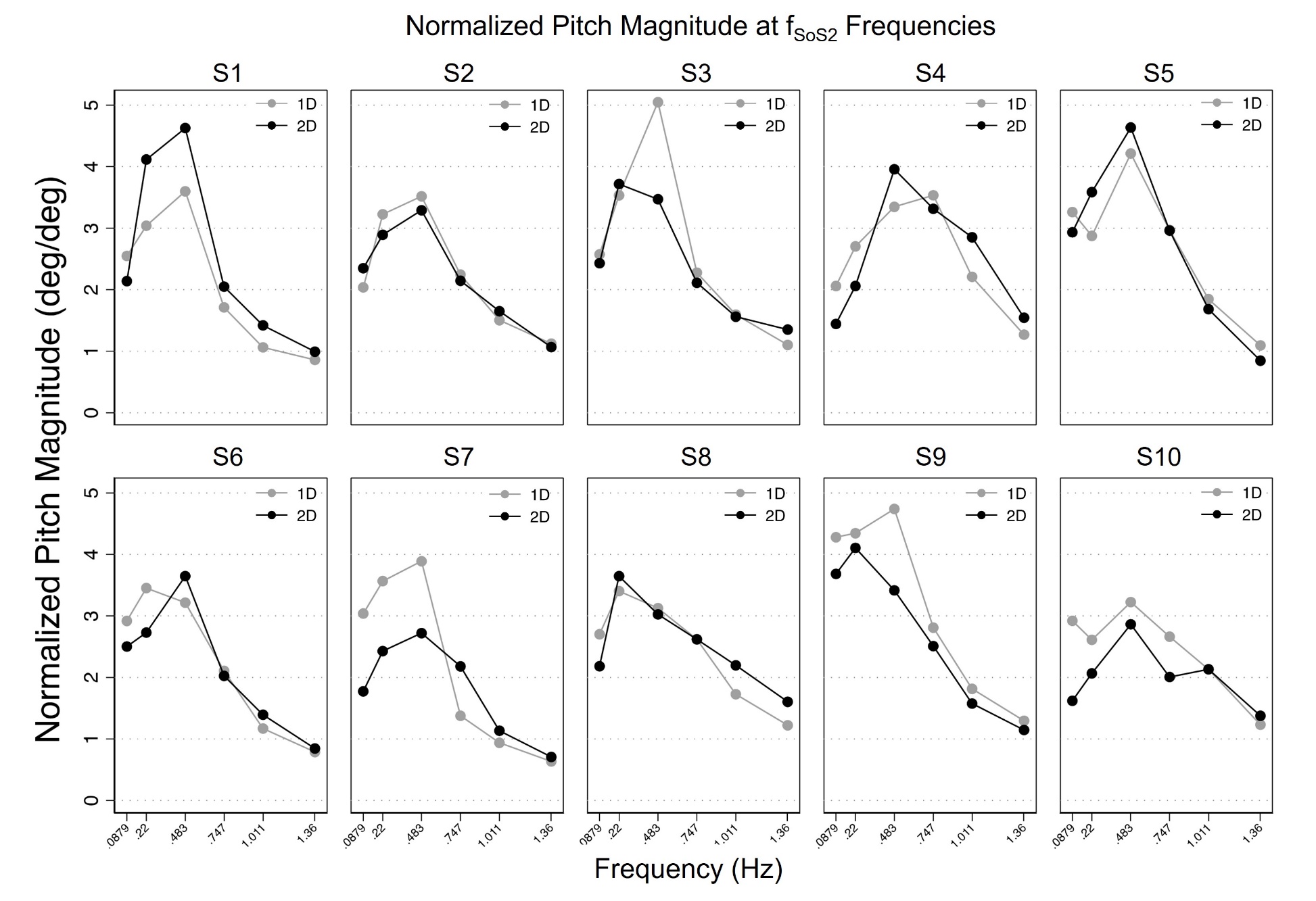
**

**Supplementary Figure 8**

**
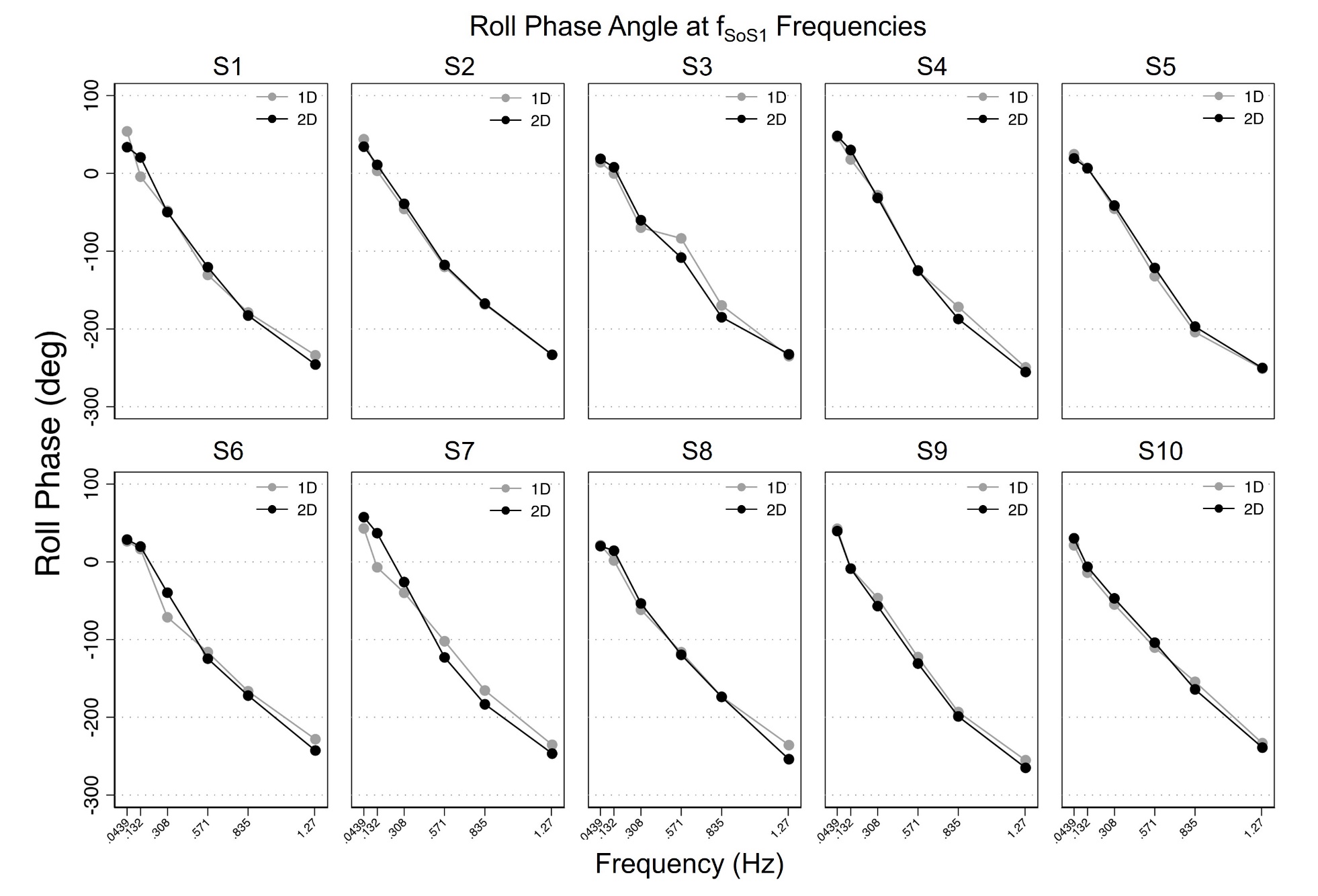
**

**Supplementary Figure 9**

**
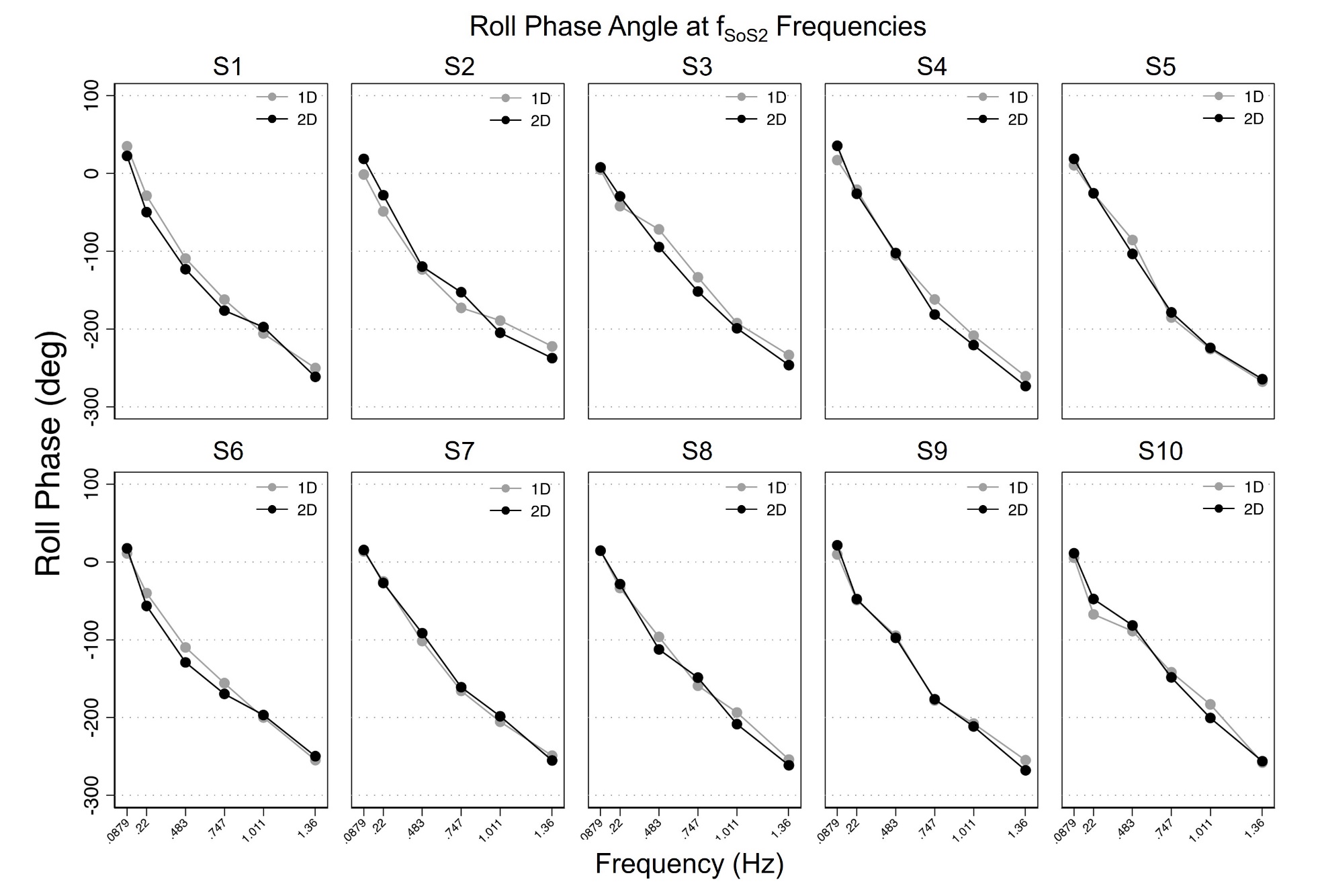
**

**Supplementary Figure 10**

**
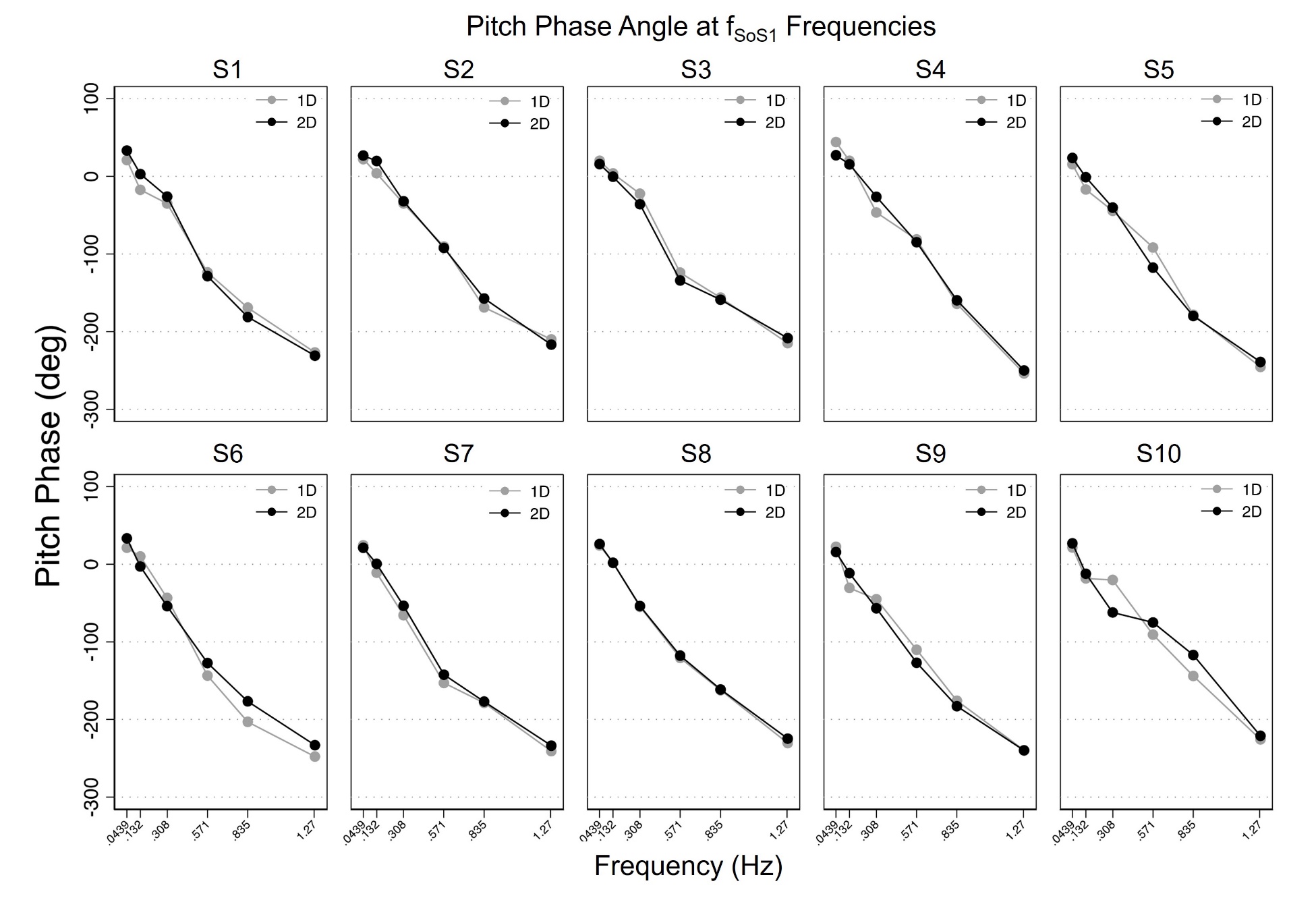
**

**Supplementary Figure 11**

**
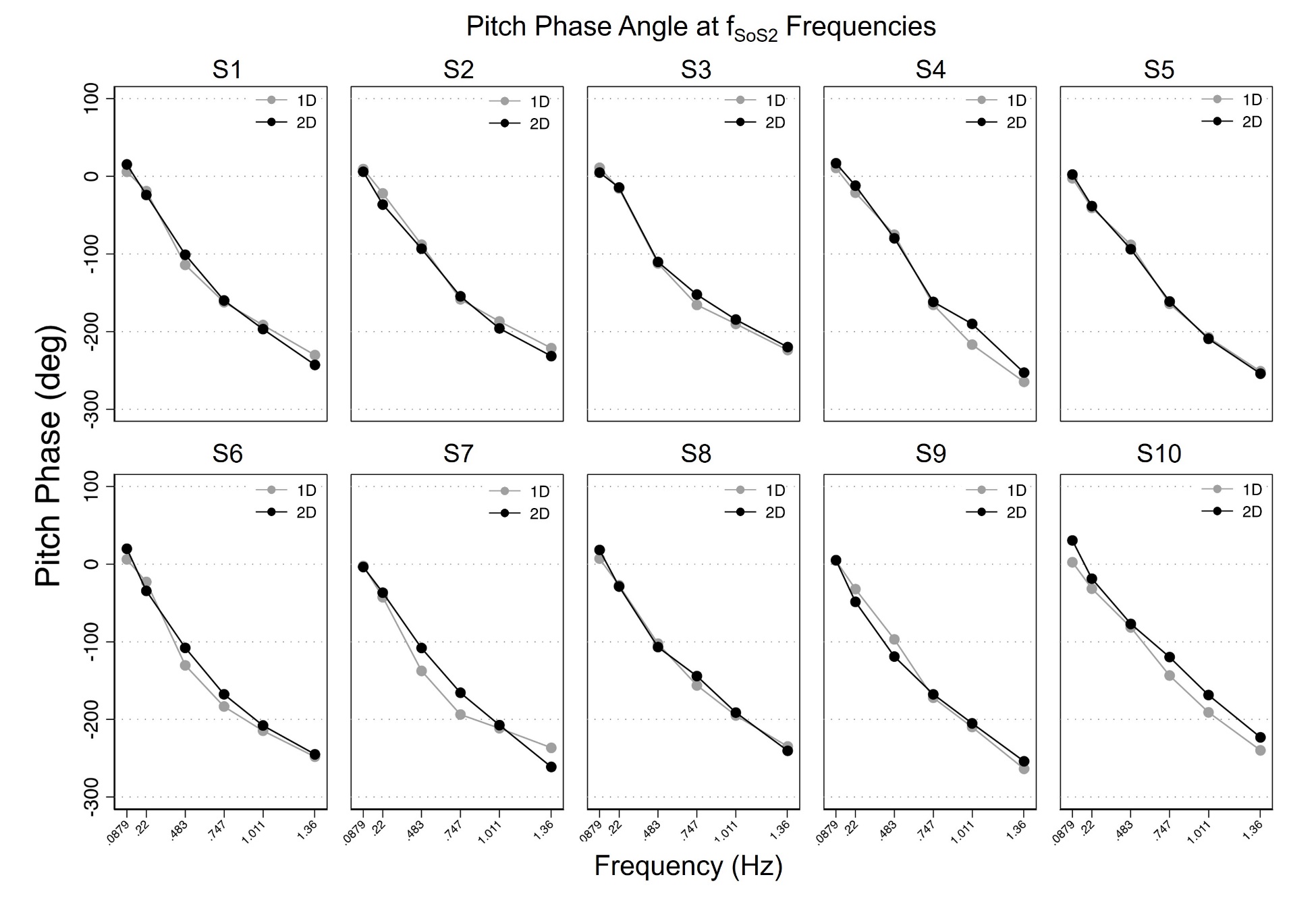
**

**Supplementary Figure 12**

**
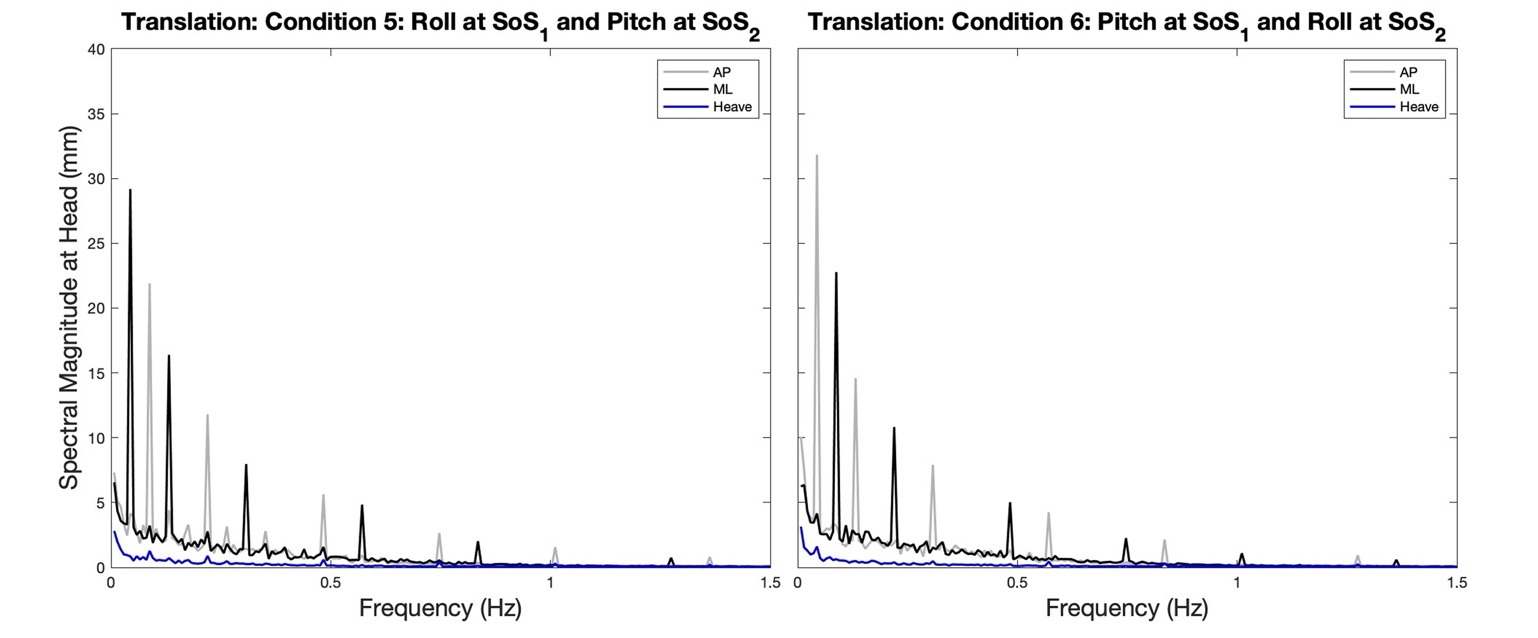
**

**Supplementary Figure 13**

**
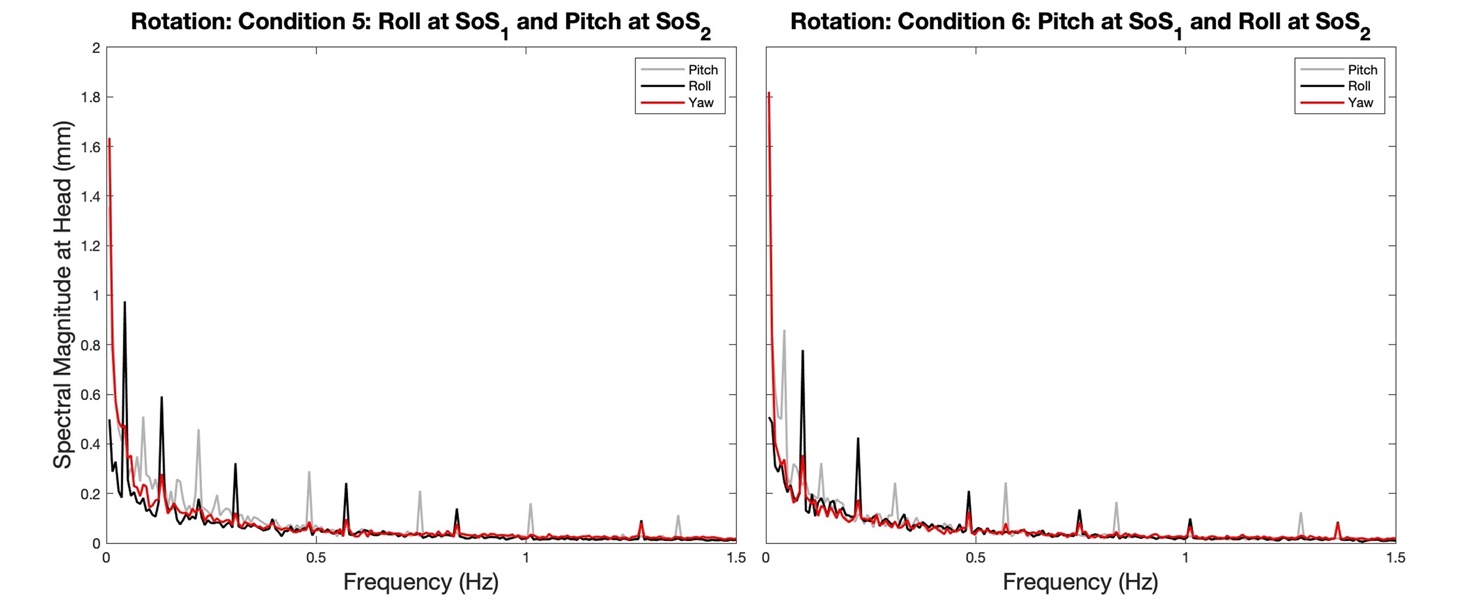
**

**Supplementary Figure 14**


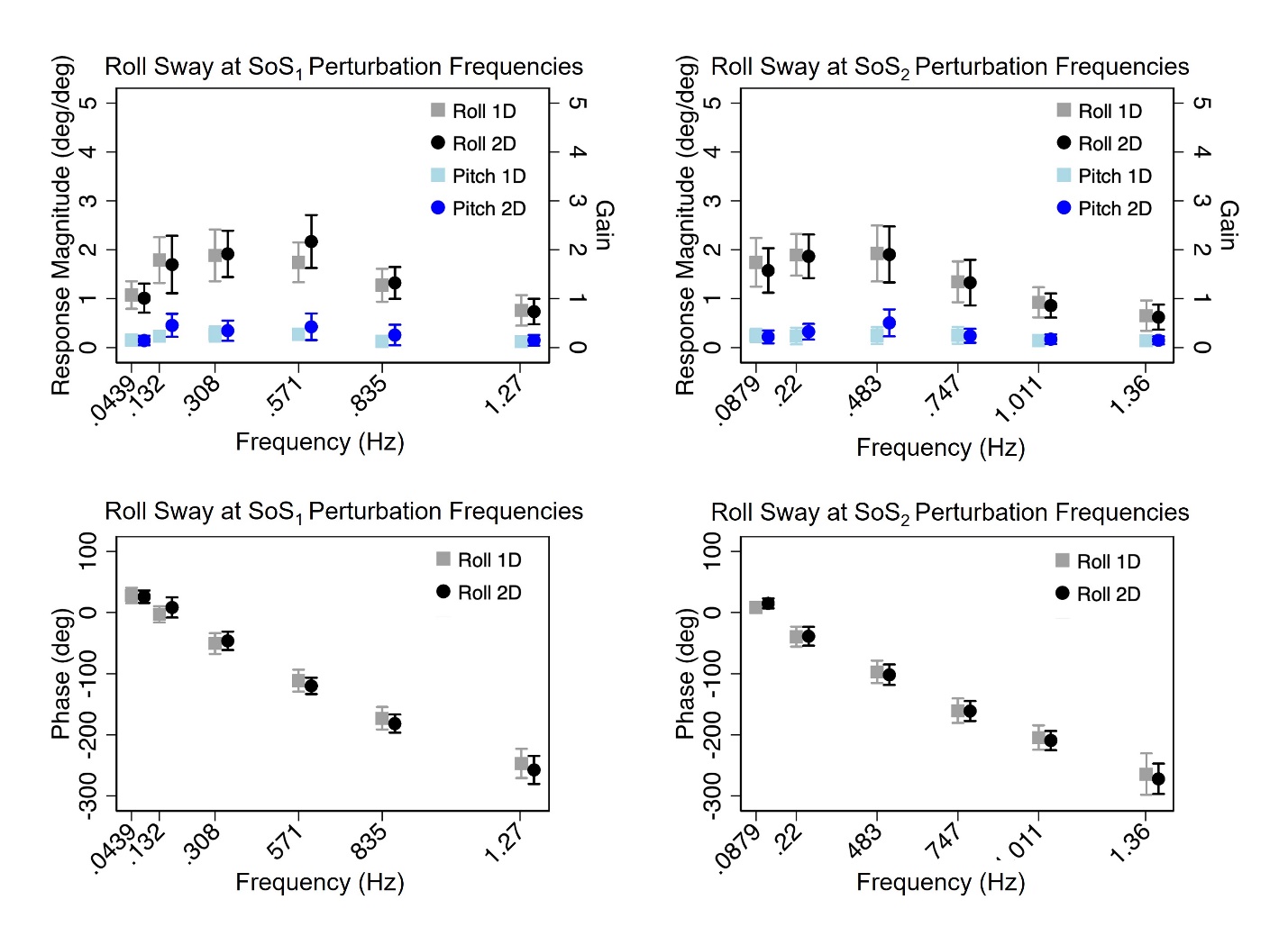


**Supplementary Figure 15**


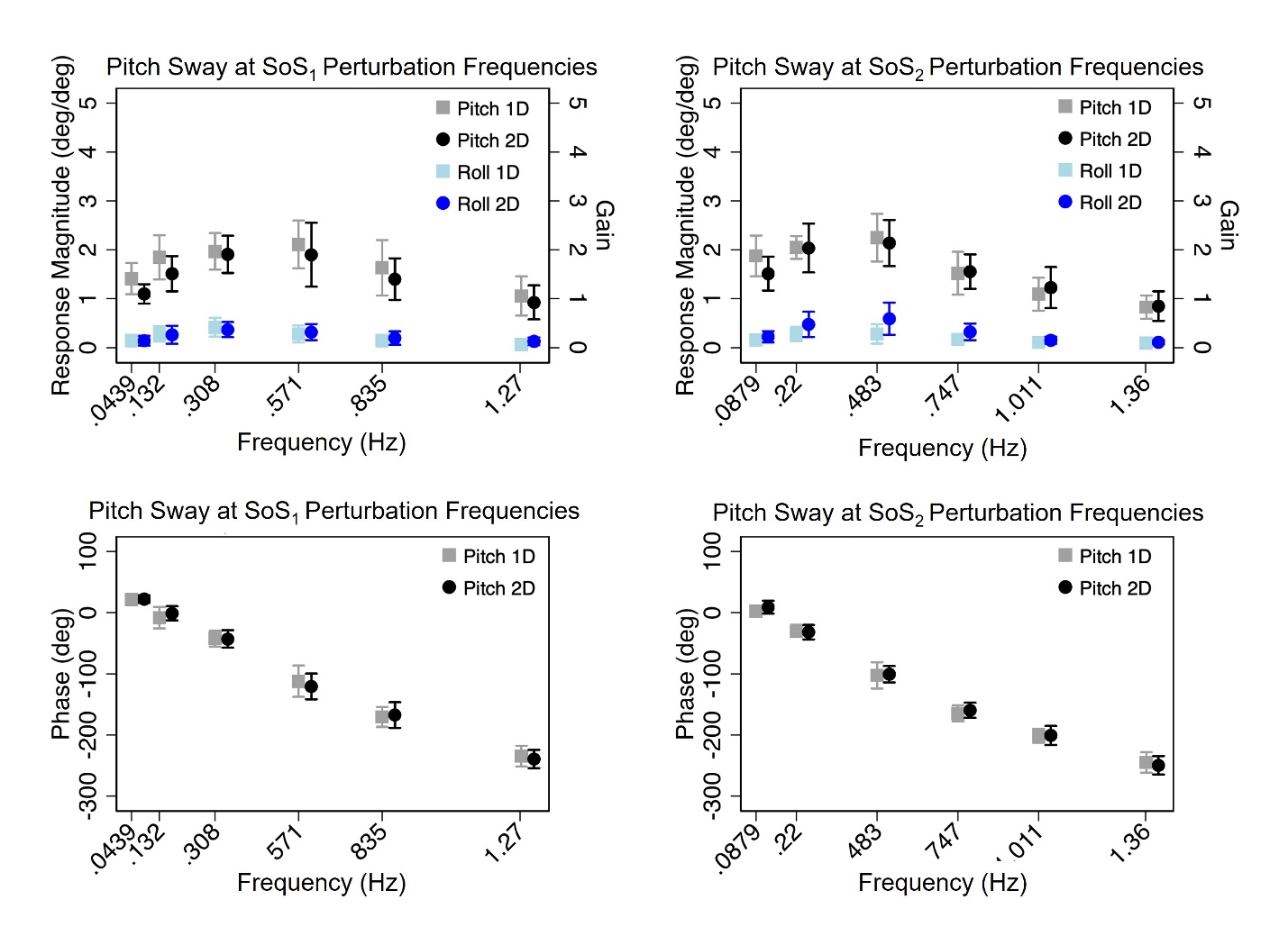


**Supplementary Figure 16**

**
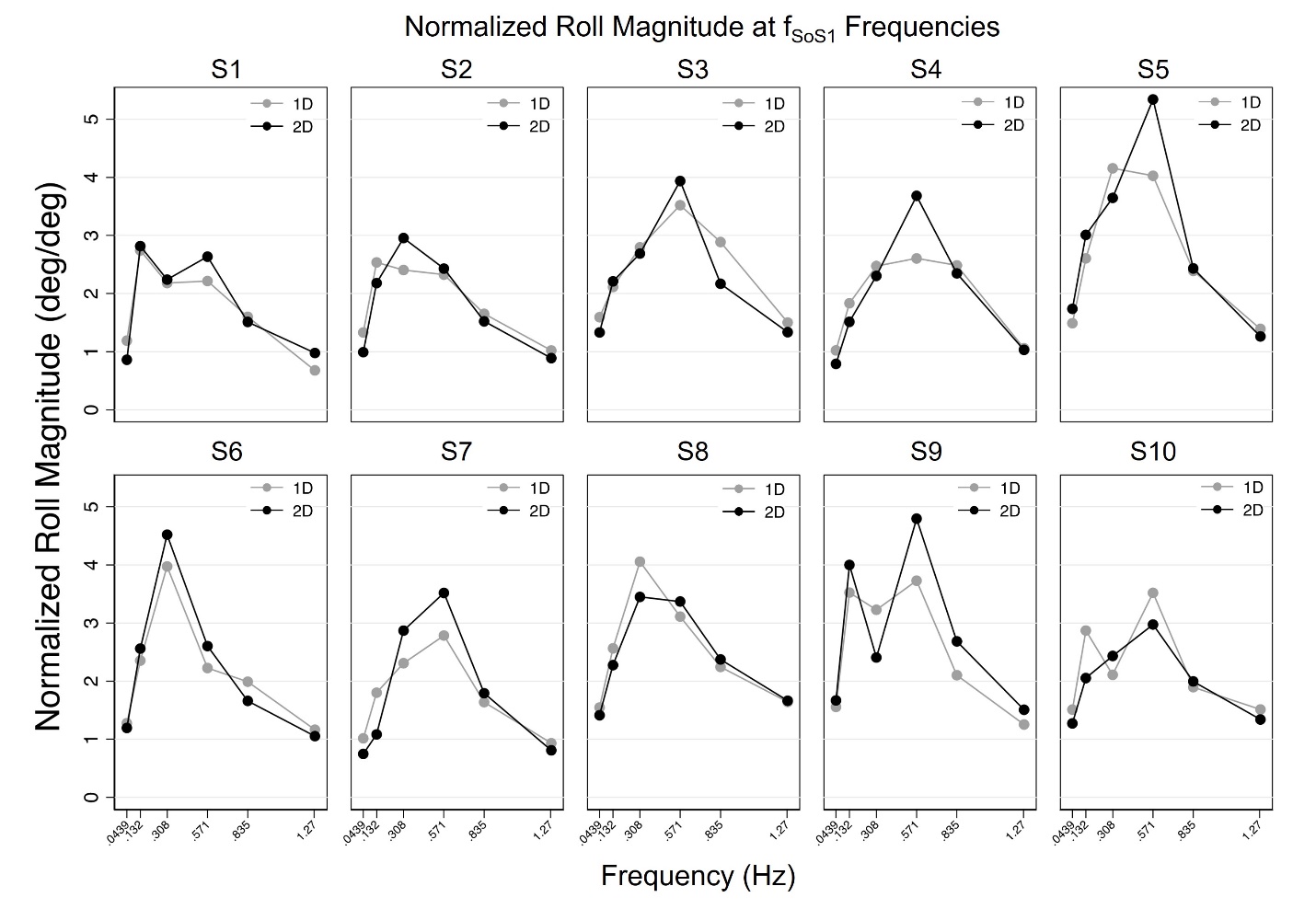
**

**Supplementary Figure 17**

**
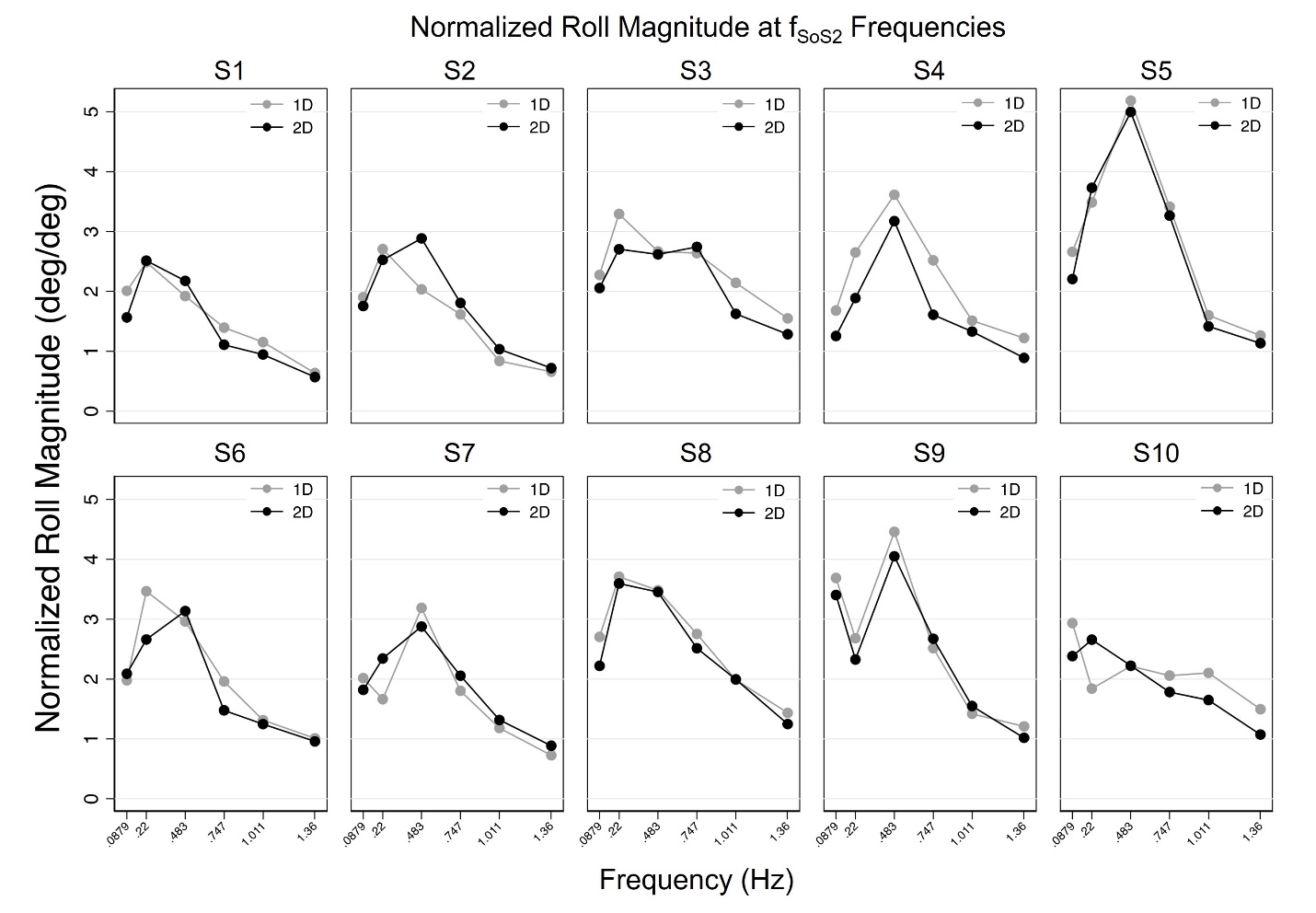
**

**Supplementary Figure 18**

**
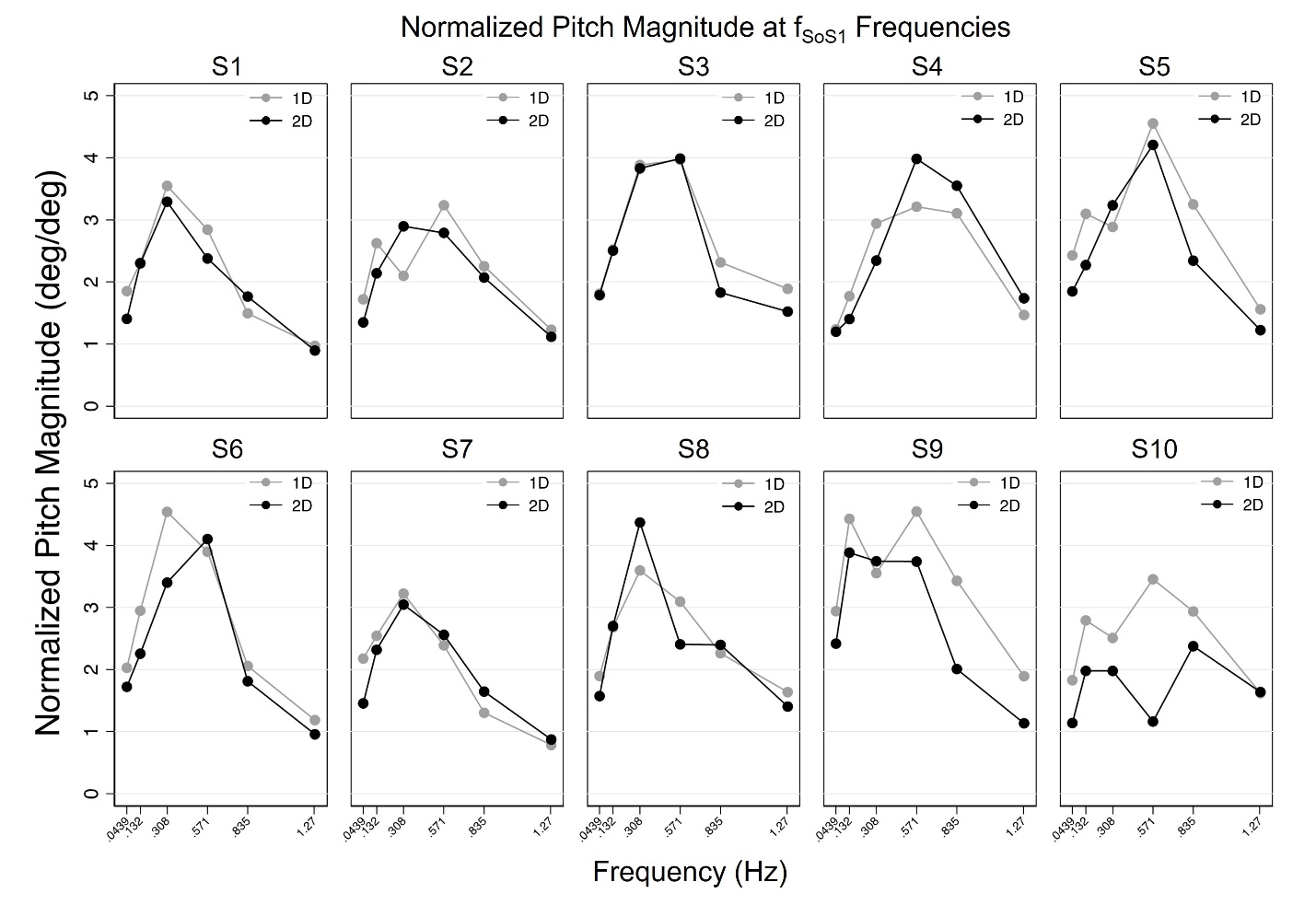
**

**Supplementary Figure 19**

**
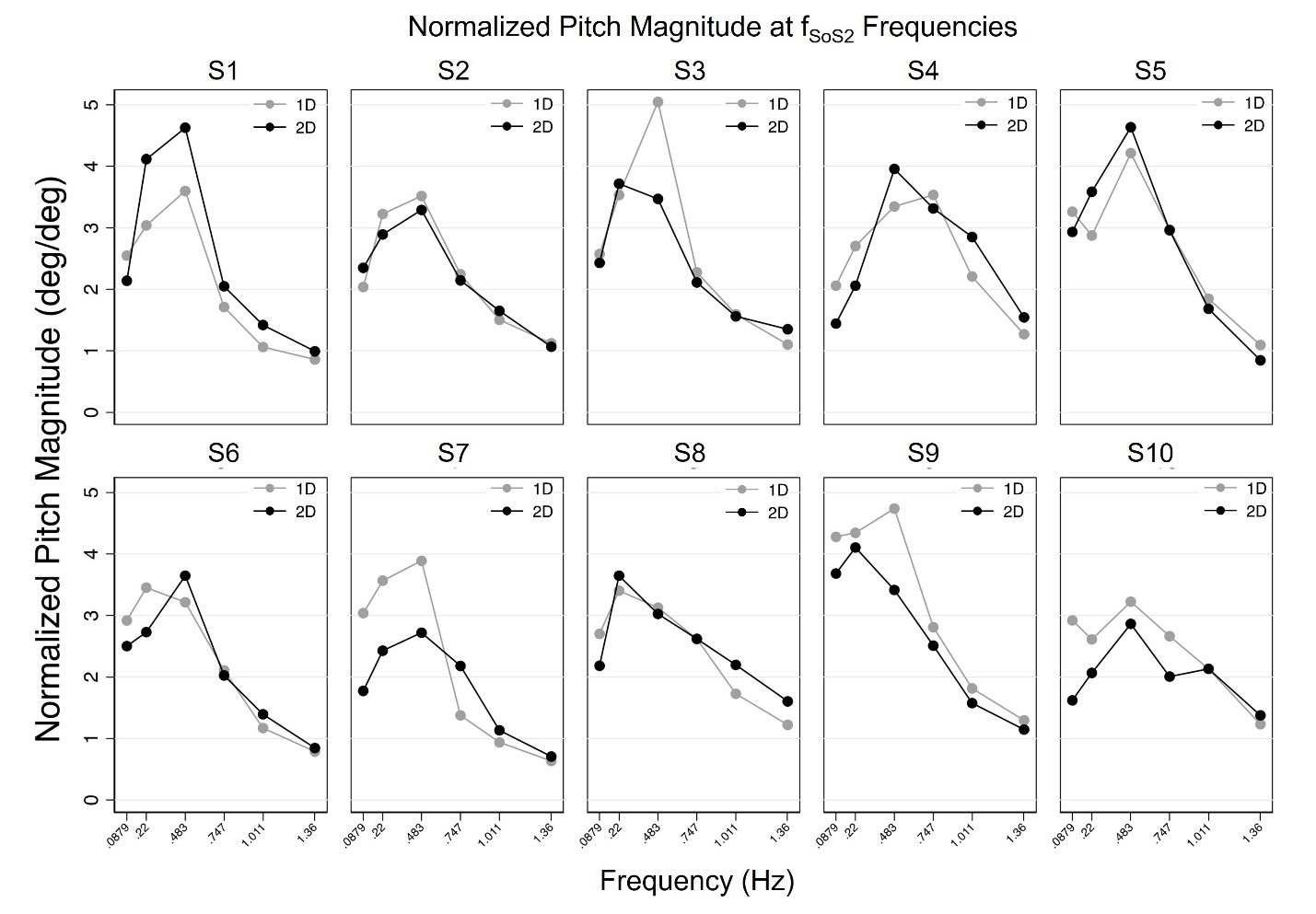
**

**Supplementary Figure 20**

**
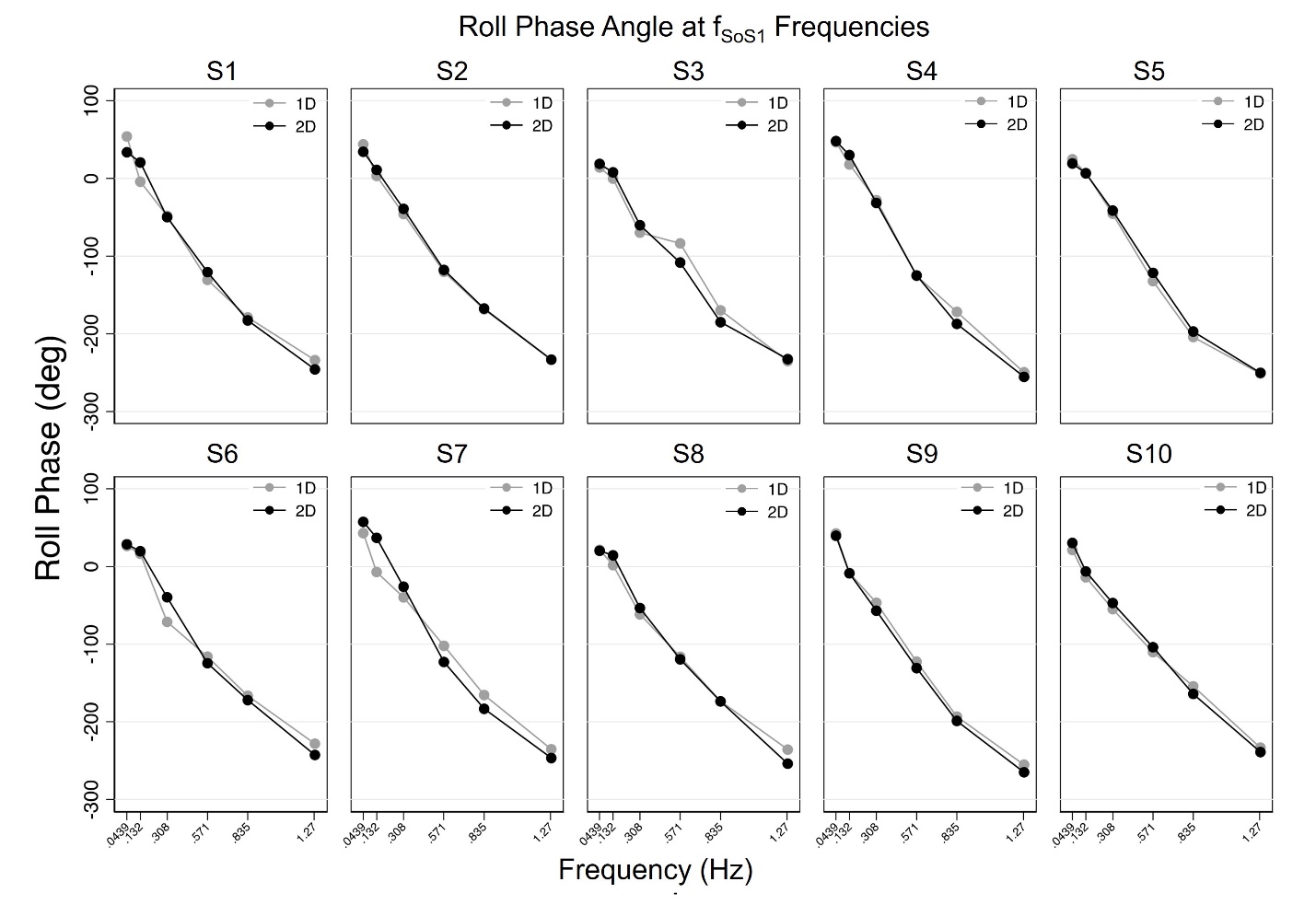
**

**Supplementary Figure 21**

**
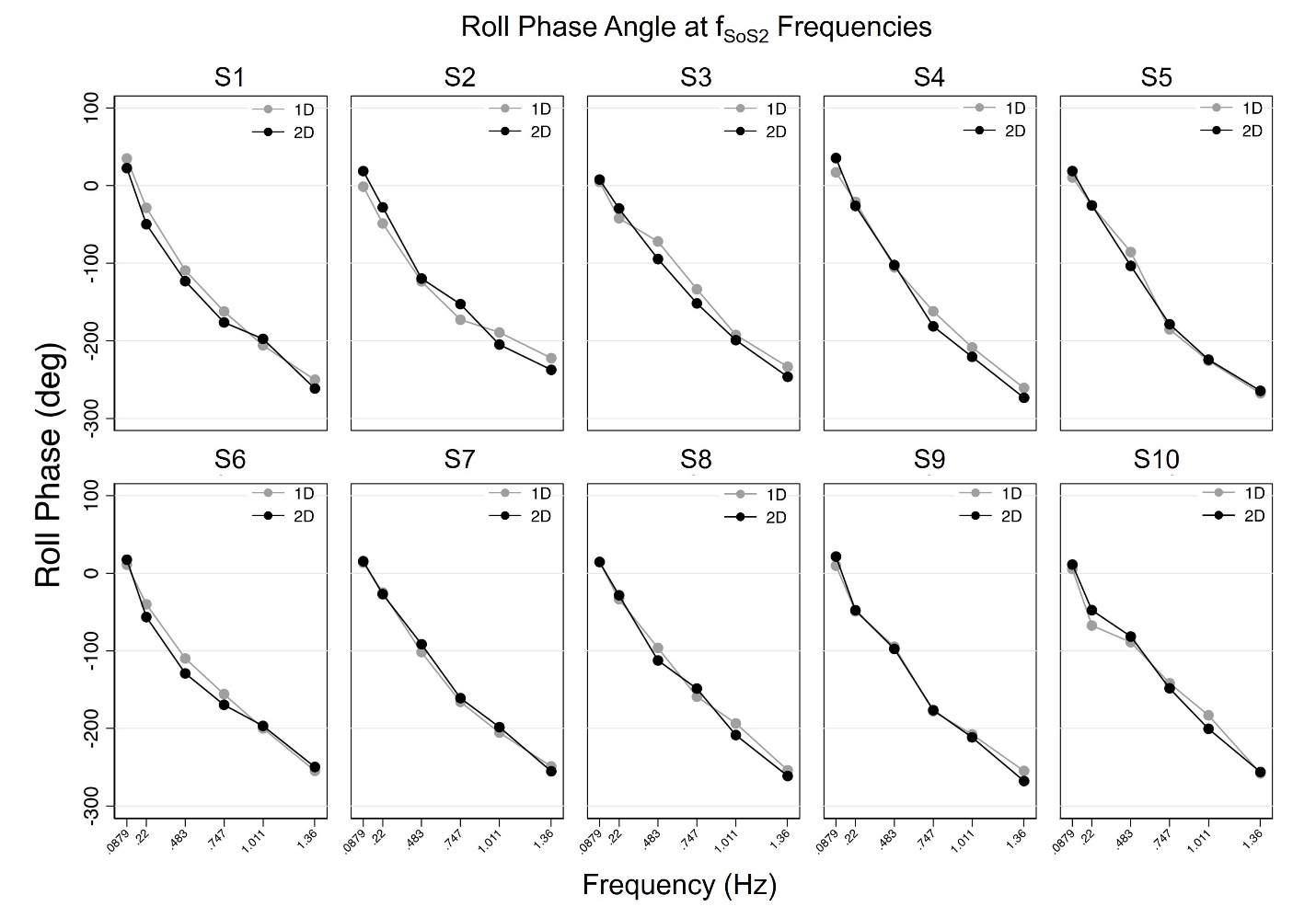
**

**Supplementary Figure 22**

**
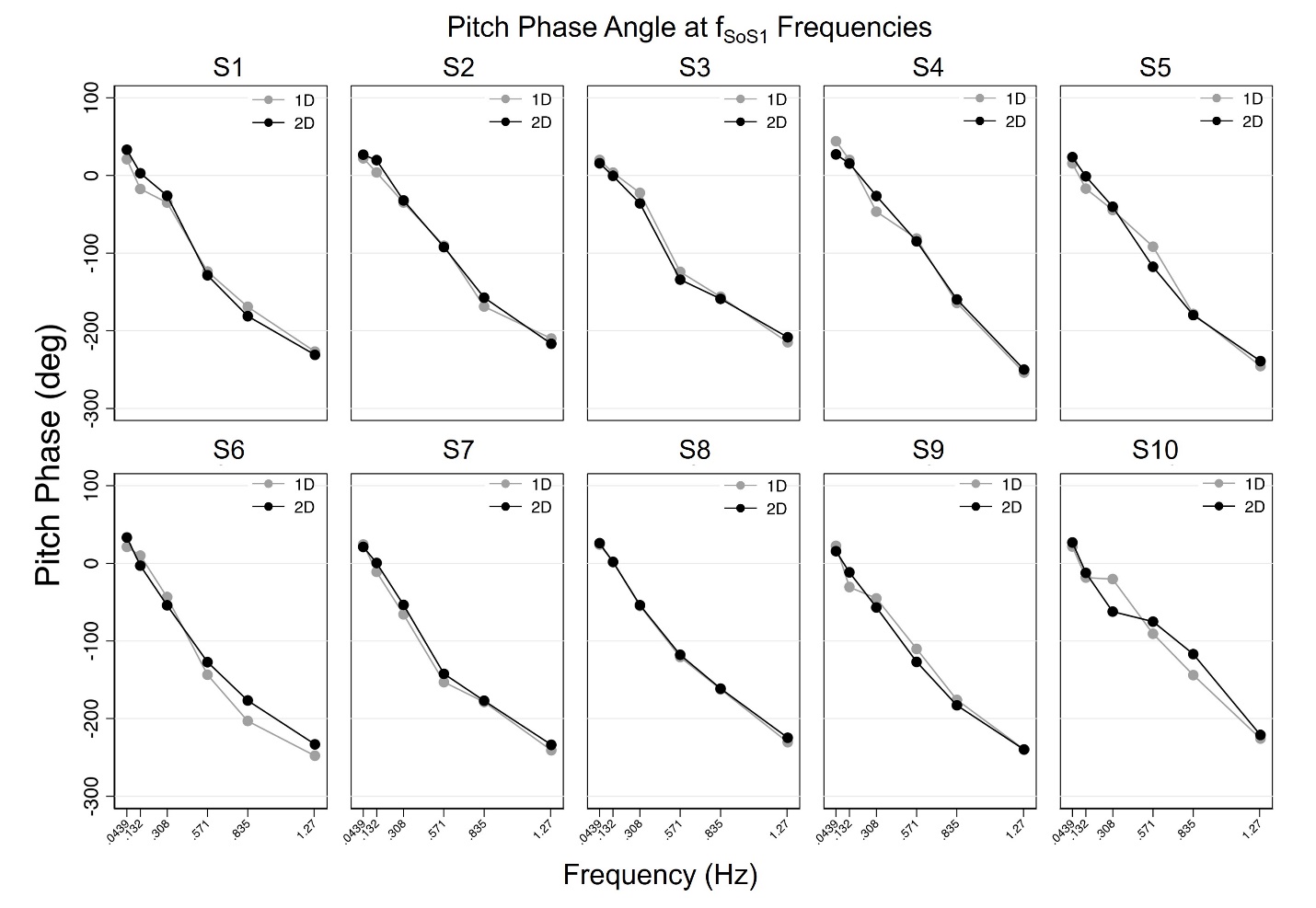
**

**Supplementary Figure 23**

**
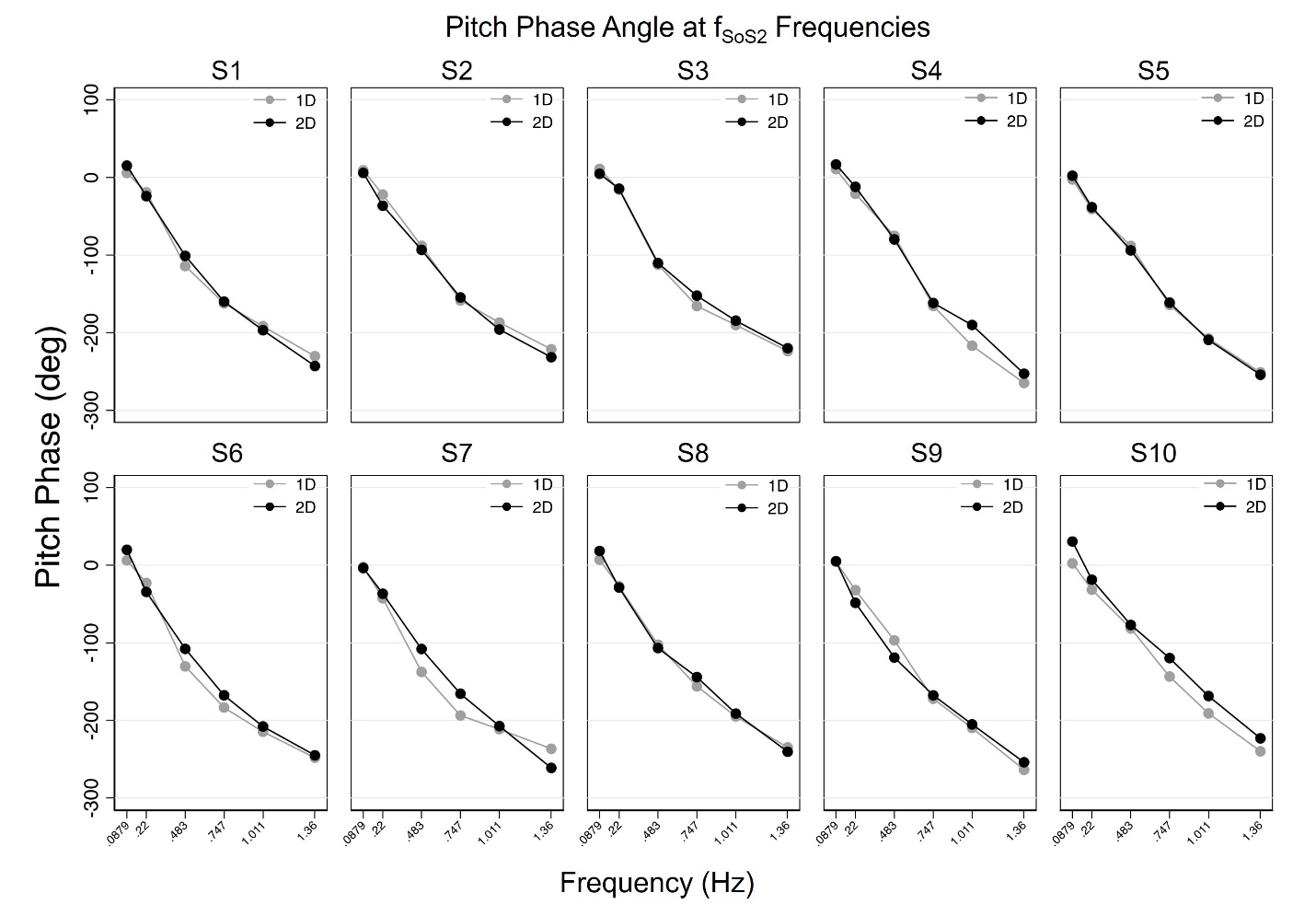
**

**Supplementary Figure 1.** The displacement (top row), velocity (middle row), and acceleration (bottom row) of the SoS_1_ (left column, red) and SoS_2_ (right column, blue) perturbation signals are shown. Each plot shows a single cycle of the perturbation. During each test condition, each of these cycles was repeated a total of seven times. The magnitude of the individual sinusoids that constitute each SoS signal were scaled to achieve nearly constant velocity (0.28˚/s) at each perturbation frequency. This yielded an SoS_1_ signal with a peak-to-peak amplitude of 1.94° and an SoS2 signal with a peak-to-peak amplitude of 1.70°.

**Supplementary Figure 2.** The spectral magnitudes of the mediolateral (ML, black) and anteroposterior (AP, grey) center of pressure (CoP) response to Condition 5 — i.e., a 2D perturbation where the roll disturbance has power at SoS_1_ frequencies, and the pitch disturbance has power at the SoS_2_ frequencies — are shown for each of the 10 participants. These spectral magnitudes were averaged across participants to yield the paper’s Figure 2A.

**Supplementary Figure 3.** The spectral magnitudes of the mediolateral (ML, black) and anteroposterior (AP, grey) center of pressure (CoP) response to Condition 6 — i.e., a 2D perturbation where the roll disturbance has power at SoS_2_ frequencies, and the pitch disturbance has power at the SoS_1_ frequencies — are shown for each of the 10 participants. These spectral magnitudes were averaged across participants to yield the paper’s Figure 2B.

**Supplementary Figure 4.** The normalized roll response magnitudes at the SOS_1_ frequencies for each individual are shown for the 2D (black) and 1D (grey) conditions.

**Supplementary Figure 5.** The normalized roll response magnitudes at the SOS_2_ frequencies for each individual are shown for the 2D (black) and 1D (grey) conditions.

**Supplementary Figure 6.** The normalized pitch response magnitudes at the SOS_1_ frequencies for each individual are shown for the 2D (black) and 1D (grey) conditions.

**Supplementary Figure 7.** The normalized pitch response magnitudes at the SOS_2_ frequencies for each individual are shown for the 2D (black) and 1D (grey) conditions.

**Supplementary Figure 8.** The phase values of the roll CoM angle at the SOS_1_ frequencies for each individual are shown for the 2D (black) and 1D (grey) conditions.

**Supplementary Figure 9.** The phase values of the roll CoM angle at the SOS_2_ frequencies for each individual are shown for the 2D (black) and 1D (grey) conditions.

**Supplementary Figure 10.** The phase values of the pitch CoM angle at the SOS_1_ frequencies for each individual are shown for the 2D (black) and 1D (grey) conditions.

**Supplementary Figure 11.** The phase values of the pitch CoM angle at the SOS_2_ frequencies for each individual are shown for the 2D (black) and 1D (grey) conditions.

**Supplementary Figure 12.** For each of the two-dimensional (2D) perturbation conditions, Condition 5 (A) and Condition 6 (B), the average (across participants) power spectra are shown for three dimensions of head mounted display (HMD) translation — AP (grey), ML (black), and heave (blue). HMD data were analyzed by removing the first cycle of data and then subtracting the mean off of each signal. The same spectral analysis described for the CoP data was then repeated using the HMD data. The AP and ML spectral peaks were found to occur at the frequencies (f_SOS1_ and f_SOS2_) of the two unique sum of sinusoids (SoS) perturbation signals (SoS_1_ and SoS_2_) that coincided with the pitch and roll motions respectively. Consistent with the absence of a heave (superior-inferior) perturbation, peaks in the heave response were minimal.

**Supplementary Figure 13.** For each of the two-dimensional (2D) perturbation conditions, Condition 5 (A) and Condition 6 (B), the average (across participants) power spectra are shown for each of the three dimensions of head mounted display (HMD) rotation/tilt — pitch (grey), roll (black), and yaw (red). HMD data were analyzed by removing the first cycle of data and then subtracting the mean off of each signal. The same spectral analysis described for the CoP data was then repeated using the HMD data. The pitch and roll spectral peaks were found to occur at the frequencies (f_SOS1_ and f_SOS2_) of the two unique sum of sines (SoS) perturbation signals (SoS_1_ and SoS_2_) that coincided with the pitch and roll motions respectively. Particularly at higher frequencies, yaw spectral peaks can also be seen at the roll perturbation frequencies.

**Supplementary Figure 14.** The mean (across participants) normalized response magnitudes (A,B) and phases (C,D) of the sway angles calculated from the head mounted display in the roll plane are shown for the 2D (black circle) and 1D (grey square) roll perturbation conditions, at each of the individual f_SoS1_ (A, C) and f_SoS2_ (B, D) frequencies. To mirror the analysis of the CoP data, roll and pitch sway angles were estimated using the HMD displacement data and the height of the HMD. The magnitude of the off-axis pitch plane response at the roll perturbation frequencies is also shown for 1D (light blue square) and 2D (blue circle) conditions. Error bars show ±1SD surrounding the mean.

**Supplementary Figure 15.** The mean (across participants) normalized response magnitudes (A,B) and phases (C, D) of the sway angles calculated from the head mounted display in the pitch plane are shown for the 2D (black circle) and 1D (grey square) pitch perturbation conditions, at each of the individual f_SoS1_ (A, C) and f_SoS2_ (B, D) frequencies. To mirror the analysis of the CoP data, roll and pitch sway angles were estimated using the HMD displacement data and the height of the HMD. The magnitude of the off-axis roll plane response at the pitch perturbation frequencies is also shown for 1D (light blue square) and 2D (blue circle) conditions. Error bars show ±1SD surrounding the mean.

**Supplementary Figure 16.** The normalized roll response magnitudes at the SOS_1_ frequencies, calculated from the head mounted display (HMD), for each individual are shown for the 2D (black) and 1D (grey) conditions.

**Supplementary Figure 17.** The normalized roll response magnitudes at the SOS_2_ frequencies, calculated from the head mounted display (HMD), for each individual are shown for the 2D (black) and 1D (grey) conditions.

**Supplementary Figure 18.** The normalized pitch response magnitudes at the SOS_1_ frequencies, calculated from the head mounted display (HMD), for each individual are shown for the 2D (black) and 1D (grey) conditions.

**Supplementary Figure 19.** The normalized pitch response magnitudes at the SOS_2_ frequencies, calculated from the head mounted display (HMD), for each individual are shown for the 2D (black) and 1D (grey) conditions.

**Supplementary Figure 20.** The phase values of the roll CoM angles at the SOS_1_ frequencies, calculated from the head mounted display (HMD), for each individual are shown for the 2D (black) and 1D (grey) conditions.

**Supplementary Figure 21.** The phase values of the roll CoM angles at the SOS_2_ frequencies, calculated from the head mounted display (HMD), for each individual are shown for the 2D (black) and 1D (grey) conditions.

**Supplementary Figure 22.** The phase values of the pitch CoM angles at the SOS_1_ frequencies, calculated from the head mounted display (HMD), for each individual are shown for the 2D (black) and 1D (grey) conditions.

**Supplementary Figure 23.** The phase values of the pitch CoM angles at the SOS_2_ frequencies, calculated from the head mounted display (HMD), for each individual are shown for the 2D (black) and 1D (grey) conditions.
